# Probabilistic Voxel-Fe model for single cell motility in 3D

**DOI:** 10.1186/2196-050X-1-2

**Published:** 2014-10-01

**Authors:** Carlos Borau, William J Polacheck, Roger D Kamm, José Manuel García-Aznar

**Affiliations:** 1 Aragón Institute of Engineering Research (I3A), Department of Mechanical Engineering, University of Zaragoza Campus Rio Ebro, 50018 Zaragoza, Spain.; 2 Departments of Biological and Mechanical Engineering, Massachusetts Institute of Technology, 77 Massachusetts Avenue, Cambridge, MA 02139-4307, USA.

**Keywords:** Cell migration, Modeling, Voxel, Finite elements, Mechanosensing, Microfluidic device

## Abstract

**Background:**

Cells respond to a variety of external stimuli regulated by the environment conditions. Mechanical, chemical and biological factors are of great interest and have been deeply studied. Furthermore, mathematical and computational models have been rapidly growing over the past few years, permitting researches to run complex scenarios saving time and resources. Usually these models focus on specific features of cell migration, making them only suitable to study restricted phenomena.

**Methods:**

Here we present a versatile finite element (FE) cell-scale 3D migration model based on probabilities depending in turn on ECM mechanical properties, chemical, fluid and boundary conditions.

**Results:**

With this approach we are able to capture important outcomes of cell migration such as: velocities, trajectories, cell shape and aspect ratio, cell stress or ECM displacements.

**Conclusions:**

The modular form of the model will allow us to constantly update and redefine it as advancements are made in clarifying how cellular events take place.

## Background

Cell motility has gained increasing prominence due to its major role in several physiological and pathological processes, e.g., morphogenesis, the inflammatory response, wound healing and tumor metastasis [1]. The way cells migrate and respond to their 3D micro-environment is a multiscale process that results from the integrated effect of the properties of the tissue extracellular matrix (ECM) and the sub-cellular constituents of the cell, mediated by the cytoskeleton (CSK). This integration process depends on multiple mechanical, chemical and biological factors [2-4]. For instance, the influence of ECM stiffness and topography (Durotaxis) has been widely investigated [5-8], showing that cells prefer to migrate toward stiffer zones of the ECM, where the focal adhesions are more stable allowing to exert higher forces [9,5,10]. Cells also respond to spatial chemical gradients (Chemotaxis) in the surrounding fluid or tissue [11,12], moving towards or away from the source of chemical variation. Variations of potential gradients (Galvanotaxis), fluid conditions and ligand adhesion gradients (Haptotaxis) are additional clues for cell migration guidance currently under study [13-17].

In fact, over the past few years, immense progress has been made in understanding cell migration, largely thanks to the active interaction between experiments, mathematical and computational modeling [18]. Due to cell motility complexity, models are taking a leading role in future developments, permitting researches to run complex biophysical and biochemical scenarios without the difficulties, time and resource consumption inherent to in vitro investigations. Many of these studies have usually focused on 2D migration, not only for simplicity but due to the lack of high quality data of cell movement in 3D. This deficiency is, however, becoming increasingly overridden especially by recent advances in microfluidic technologies which allow high resolution imaging and provide enormous flexibility in controlling the critical biochemical and biomechanical factors that influence cell behavior [19,20].

Hence, the number of 3D migration models has been gradually increasing, although focused on different aspects of cell motility. Some of them predict individual cell migration [21-23], while others simulate collective behavior [24,25]. In addition, different levels of detail are described, with time and length-scales varying significantly. Rangarajan and Zaman [18] reviewed some type of models according to their main assumptions and grouped them in: (i) Force based dynamics models, (ii) Stochastics, (iii) Multi-Cell Spheroid migration, (iv) Monte Carlo studies. In the former ones, migration dynamics are accounted for by the traction forces at both the front and rear end of the cell and forces due to viscous drag and cell protrusion into the ECM [21]. Imbalances of these forces produce cell migration. The drawback of these models is that they only predict migration of single cells, not taking into account changes in cell shape or ECM properties due to degradation. On the other hand, stochastic models of persistent random walks are able to predict population behavior [26,22]; however, they don’t include dynamic effects such as traction or drag, nor incorporate the ECM properties. Multi-cell spheroid migration models are mainly based on pressure gradients produced by proliferation and death of cells [27]. Combining random walks, pressure and chemotactic activity of cell aggregates make these models suitable to study tumours, but fail to take into account mechanical cues such as ECM density, porosity or stiffness. Finally, Monte Carlo models using square lattices and a set of simple rules allow faster simulations thus providing long-term migration patterns [28,29]. The main handicap is the qualitative nature of the studied parameters such as cell-matrix interface, cell polarization or ECM mechanical effects.

In this work we develop a probabilistic FE 3D migration model for individual cells, presenting features from several of the previous mentioned types. With this model we are able to study the influence of multiple external stimuli (namely ECM stiffness, chemistry, flow and boundary conditions) estimating important features of cell migration such as: velocities, trajectories, cell shape and aspect ratio, cell stress, ECM displacements etc. Finally, we qualitatively and quantitatively compare our results with recent experiments, finding a good agreement and showing the consistency and the adaptability of the model to simulate different conditions.

Therefore, the final goal of this work is to provide a versatile and modular tool capable of predicting migration phenomena under different environmental stimuli, reducing the number and helping in the design of new experiments.

## Methods

The macroscale conditions evaluated at the cell surface influence its behavior, changing its morphology and thus determining the migration. With this in mind, several approaches could have been valid to model cell motility in 3D or other related phenomena, such as the classical FEM [30] or the more specific surface finite element method (SFEM) [31]. However, for simplicity and due to the advantages of lattice-based models, a FE approximation using voxels was chosen for the simulations as described below.

### Numerical implementation

This work describes a probabilistic voxel-FE model for 3D migration at the cell-scale level, influenced by chemical and flow conditions coming from a microfluidics simulation and the mechanical conditions of the environment. For this purpose, the ECM as well as the embedded cell are discretized with voxels, each of them corresponding with the component of a three-dimensional mathematical matrix of data (***M***) which contains relevant information for the simulation. For instance, ***M*** stores the centroid of each voxel and whether a specific component corresponds to ECM or cell, therefore determining its mechanical properties. Also, this matrix ***M*** includes the flow and chemical conditions interpolated from the microfluidic simulation, therefore containing all the necessary input factors used in the probability/cell-dynamics functions.

**Figure 1:**
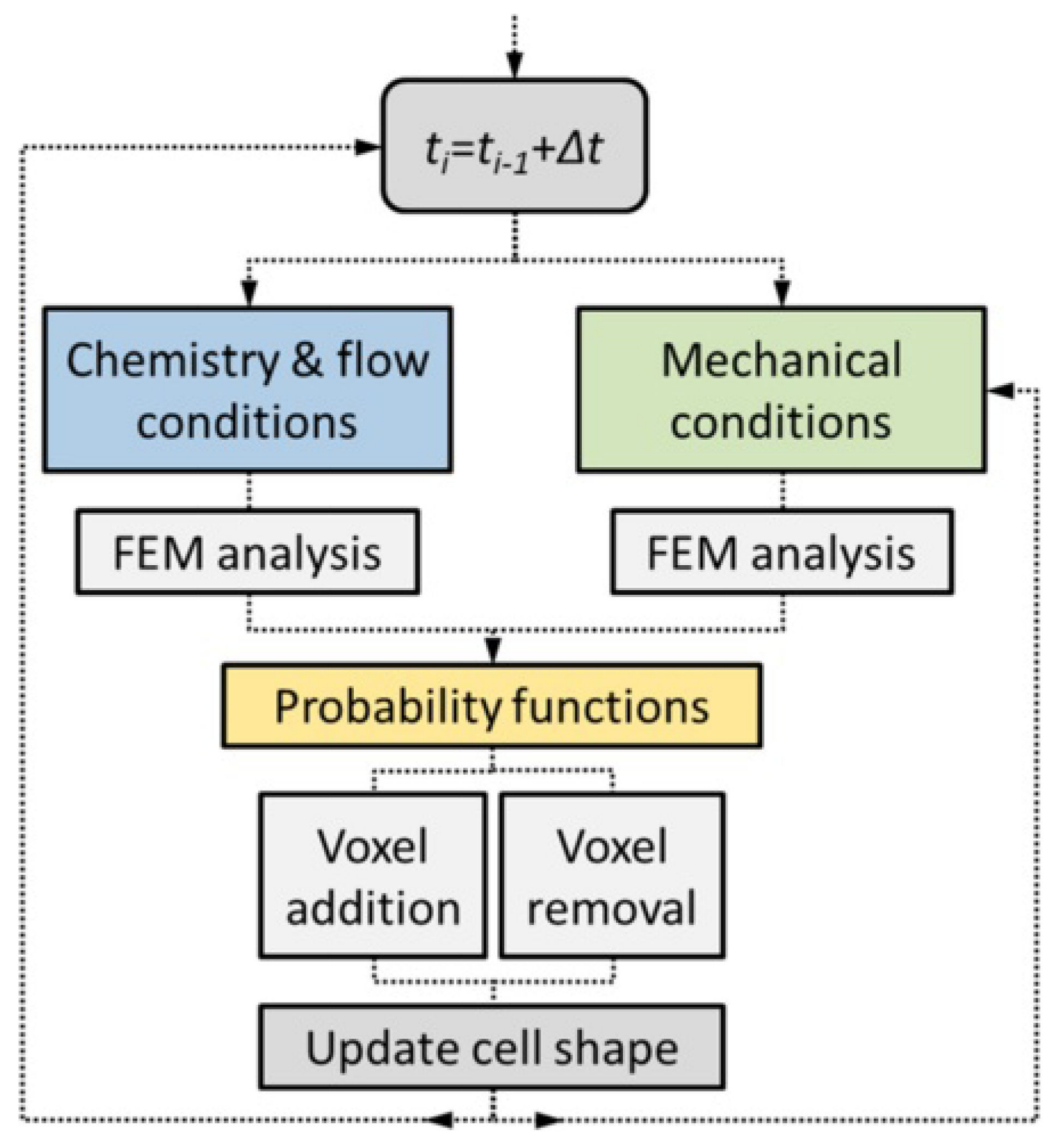
Scheme of the iterative loop At each temporal step the fluid chemical and mechanical conditions determine the probability of adding/deleting voxels to/from the cell. At the end of the step, the cell shape is updated. Note that to save computational time, chemistry and flow conditions are considered constant through the simulation, performing the corresponding FE analysis only once at the beginning and not at each time step.

At this point it is useful to present the iterative scheme (Figure 1) which can be described as follows: (i) mechanical, chemical and flow conditions are collected from the corresponding FE analysis. These data serve as input for (ii) the cell-dynamics functions which determine the probability of whether an ECM-type voxel becomes a cell-type voxel or vice versa. (iii) A random-number generator checks the probability corresponding to each voxel so the cell shape is updated. Note that only ECM voxels in contact with the cell may become cell, and that only voxels of the cell surface may become ECM. It is also important to clarify, that the cell-voxel distribution (cell shape) is essential for the mechanical analysis since the cell forces are the only ones taken into account. Hence, the mechanical problem is computed at each step, whereas the fluid chemical analysis is computed only once at the beginning. This choice saves computational time and it is justified by the fact that the cell volume is much smaller than the problem domain (collagen). Therefore, assuming steady state at the microdevice, it is considered that the cell shape does not affect the fluid-chemical analysis carried out in the first step. Nevertheless, to test this simplification, a specific fluid-chemical simulation with a random cell shape embedded in a porous matrix was performed. The results confirmed that its effects on the stationary solution are negligible (Additional file 1). Hence, the fluid-chemical conditions are considered constant through the simulation.

### Mathematical modeling

So far the general iteration scheme has been described, but not how the fluid chemical and mechanical problems are solved. As explained below, these problems are computed separately although interacting via changes in cell shape and position which depend, through the probability functions, on several environmental input factors as described in next section.

#### Modeling chemotaxis and flow through a porous medium

**Figure 2:**
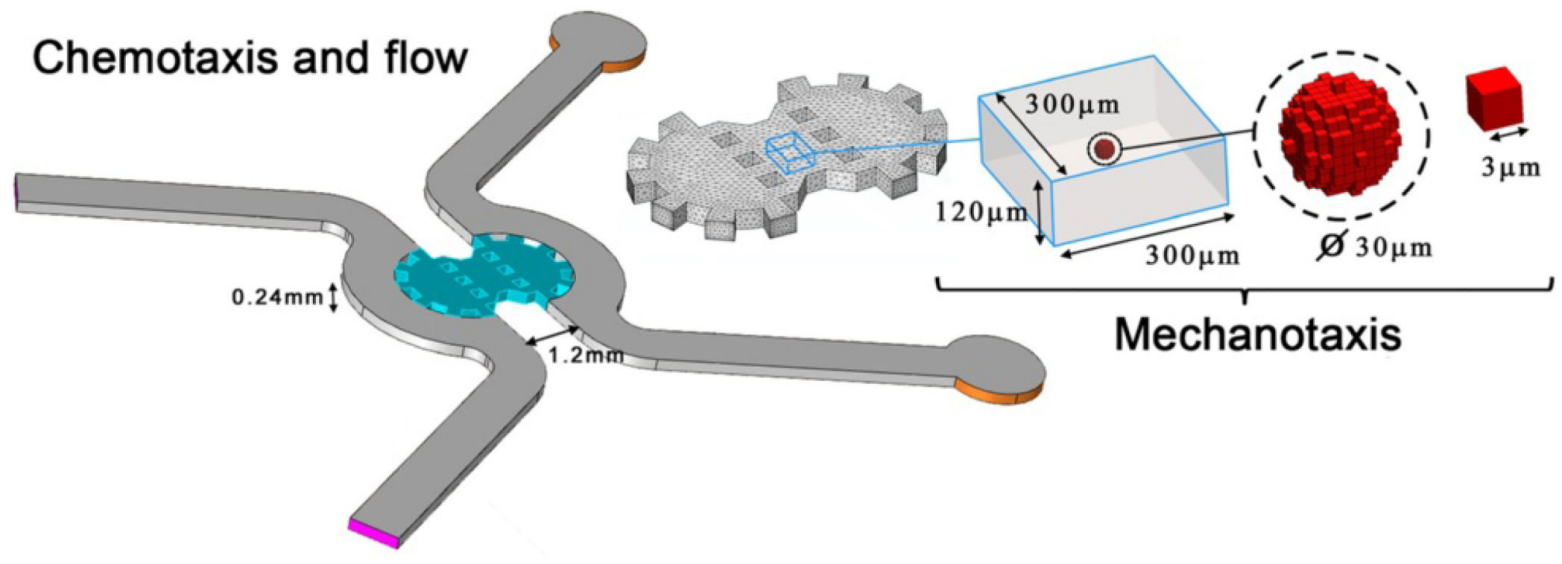
Geometry of the microfluidic device and details of domain and cell mesh Left: two channels (gray) are separated by collagen I gel (cyan). Pressure and chemical gradients are established between inlet (purple) and outlet (orange) boundaries. A box-like domain (right) is taken from the central part of the gel to simulate the mechanical analysis and the cell migration. This domain is discretized with voxels of 3 μm, some of them considered cell-voxels and forming an initially spherical shape of about 30 μm of diameter embedded in the ECM to perform the mechanical and migration simulation.

A complete 3D microfluidic device is simulated, the geometry and boundary conditions of which are taken from a recent experiment [13]. This device consists of two channels separated by a region containing single cells suspended in collagen I gel (Figure 2). Applying a hydrostatic pressure gradient across the gel region a consistent flow field is generated. In addition, different chemical concentrations are established up and downstream, generating a linear chemical gradient, which, although difficult to obtain experimentally, is useful in the simulations to test the model. Note that this gradient is different from the gradient in the experiments [13], which is autocrine and arises from cells secreting chemoattractant. Finite element software (COMSOL Multiphysics) is used to compute the flow through collagen and the transport of diluted species:

(1) $$\frac{\partial c}{\partial t}+\nabla [uc-D\nabla c]=R$$

where *c* is the concentration of the diluted species, *D* is the diffusion coefficient, *R* is a production or consumption rate expression (for simplicity, 0 in the simulations) and **u** is the solvent velocity field.

The flow in porous media is governed by a combination of the continuity equation and momentum balance equation, which together form the Brinkman equations:

(2) $$\frac{\partial }{\partial t}(e_{p}\rho )+\nabla \cdot (\rho u)=Q_{\mathrm{br}}$$

(3) $$\frac{\rho }{e_{p}}\left(\frac{\partial u}{\partial t}+(u\cdot \nabla )\frac{u}{e_{p}}\right)=-\nabla P+\nabla \left(\frac{\mu }{e_{P}}\left(\nabla u+{\left(\nabla u\right)}^{T}\right)-\frac{2}{3}\frac{\mu }{e_{p}}(\nabla u)I\right)-\left(Q_{\mathrm{br}}+\frac{\mu }{\kappa }\right)u+F$$

In these equations, *μ* denotes the dynamic viscosity of the fluid, **u** is the velocity vector, *ρ* is the density of the fluid, *P* is the pressure, *e*_p_ is the porosity, *κ* is the permeability of the porous medium, and *Q*_br_ is a mass source or mass sink (which has been considered 0 in all the simulations). Influence of gravity and other volume forces can be accounted for via the force term **F**, although they are neglected, as well as the inertial term ((**u** · ∇)**u**/*e*_p_), in the current simulations. With all this, and assuming incompressible flow (∇**u** = 0), equations 1,2 and 3 are drastically simplified. Values of these main parameters are listed in Table 1.

Since the purpose of this work is to study the migration of a single cell, which volume is negligible in comparison with the whole microdevice domain, the steady state simulation is performed only once, not considering the embedded cell body. Then the results from a central box-like region are extracted to compute the mechanical analysis and the cell migration (Figure 2, right). Note that no chemical species secreted by the cell are considered here for simplicity. Hence, the chemical concentration and flow direction at each point of the box-like domain remain unalterable regardless cell position in the subsequent steps of the migration simulation. As pointed before, the effect of a 3D body embedded in the centre of the gel is analysed to support this assumption, finding that its influence was practically null except at points very close to the body surface (Additional file 1).

#### Modeling mechanotaxis

The steady-state solution from small box-like domain the fluid simulation is extracted and interpolated into an organized mesh and stored in ***M***. Specifically the domain is discretized with voxels of 3 μm, some of them assigned to model cell behavior (from now called cell-voxels) and forming an initially spherical-like shape embedded in the ECM (Figure 2, right). This size is adequate to roughly mimic cell-like morphologies without increasing too much the computational cost. Smaller sizes, that would improve the accuracy of the cell surface, would produce an excessively refined mesh of the domain which would lead in turn to heavier and slower simulations. For simplicity, the ECM is considered linear elastic, whereas cell-voxels have their own mechanical properties.

**Figure 3:**
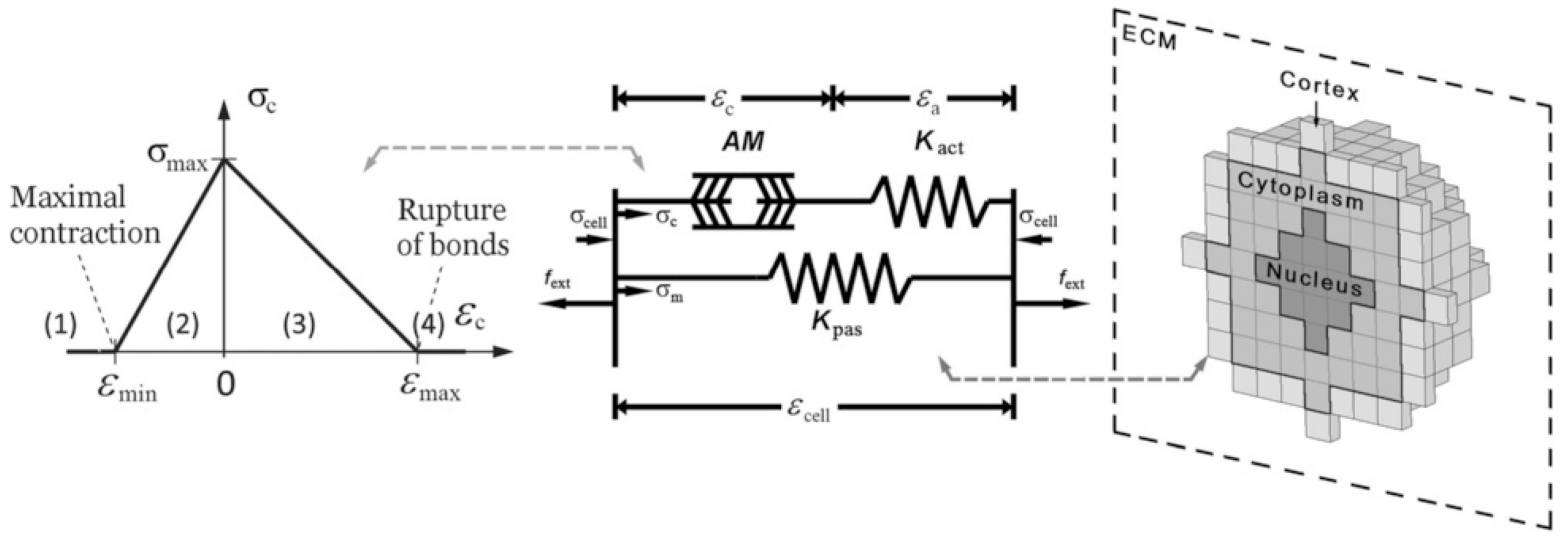
Mechanosensing scheme for 3D and different cell parts Cell material is modeled using two springs in parallel representing the actin stiffness (*K*_act_) and the passive components (*K*_pas_) of the cytoskeleton, in series with an active actuator representing the myosin machinery (*AM*) Left plot shows the stress exerted by the *AM* as a function of the sliding between actin filaments and myosin arms (*ε_c_*). Cell-voxels (right) are divided in three zones: cortex (light gray), cytoplasm (medium gray) and nucleus (dark gray). The nucleus plays only a passive role and is modeled as an elastic material. The cortex and cytoplasm, however, present a contractile behavior depending on ECM stiffness, following the mechanosensing model.

In similar fashion to previous works [22], the *mechanosensing* behavior of each cell-voxel is simplified to two springs representing the actin stiffness (*K*_act_) and the passive components (*K*_pas_) of the cytoskeleton, and an active actuator representing the myosin machinery (*AM*), each of them assumed to independently act in the x,y,z directions (Figure 3). The stress exerted by this actuator depends upon the sliding between actin filaments and myosin arms (*ε*_c_), which is limited by a maximum contraction parameter (*ε*_min_). This sliding depends in turn on the cell strain (*ε*_cell_) and therefore on the ECM stiffness. Hence, cell stress transmitted to the matrix by each voxel in each direction “*i*” can be expressed as a function of cell strain:

(4) σcelli={Kpasεcelliεcelli<εminKactσmaxKactεmin−σmax(εmin−εcelli)+Kpasεcelliεmin<εcelli<(σmax∕Kact)KactσmaxKactεmax−σmax(εmax−εcelli)+Kpasεcelli(σmax∕Kact)<εcelli<εmaxKpasεcelliεmax<εcelli}

The main difference with respect to the approach used in [22], is that the polarization term is not explicitly included in the stress tensor (which is now isotropic), since the polarization direction emerges from the cell morphology. Also note that in the probability functions (explained in next section) only one value of stress is used, in particular the volumetric stress of each voxel $\left(\sigma_{v}=\left(\sigma_{\text{cell}}^{x}+\sigma_{\text{cell}}^{y}+\sigma_{\text{cell}}^{z}\right)∕3\right)$. In the present model, three different zones of the cell body are considered: cortex, cytoplasm and nucleus (Figure 3, right). In a first approach, the only difference between the cortex zone and the cytoplasm is the exertion of higher stress, therefore assigning higher *σ*_max_ to the cortex-voxels (2.5 kPa compared with 1.5 kPa at the cytoplasm). This is a first approximation to reflect the higher forces exerted by the cells at their perimeter, mainly due to the increased presence of focal adhesions [32-36]. On the other hand, the nucleus presents no contractile behavior, so only its passive resistance (*K*_pas_) is considered (acto-myosin actuator and actin branch are therefore disabled in the corresponding voxels). All of these parameters are listed in Table 1.

The mechanical problem is computed at each step, taking into account the redistribution of voxels belonging to each zone of the cell or to the ECM. To solve that, a user-subroutine of the software ABAQUS together with a MATLAB script are employed. Once the FE subroutine computes the mechanical equilibrium at each step, the script comes into action to compute the probabilities of voxel addition/removal according with the mechanical, flow and chemical conditions. In this process, the cell shape is updated as well as all the necessary variables of ***M***. These data act as an input for the FE subroutine in the next step, repeating the process until the end of the simulation. Note that the mechanical analysis only corresponds to the cell-matrix interactions, and not to the flow-ECM or flow-cell interactions which are not considered in this first approach.

#### Probability functions: external stimuli and cell dynamics determine cell migration

**Figure 4:**
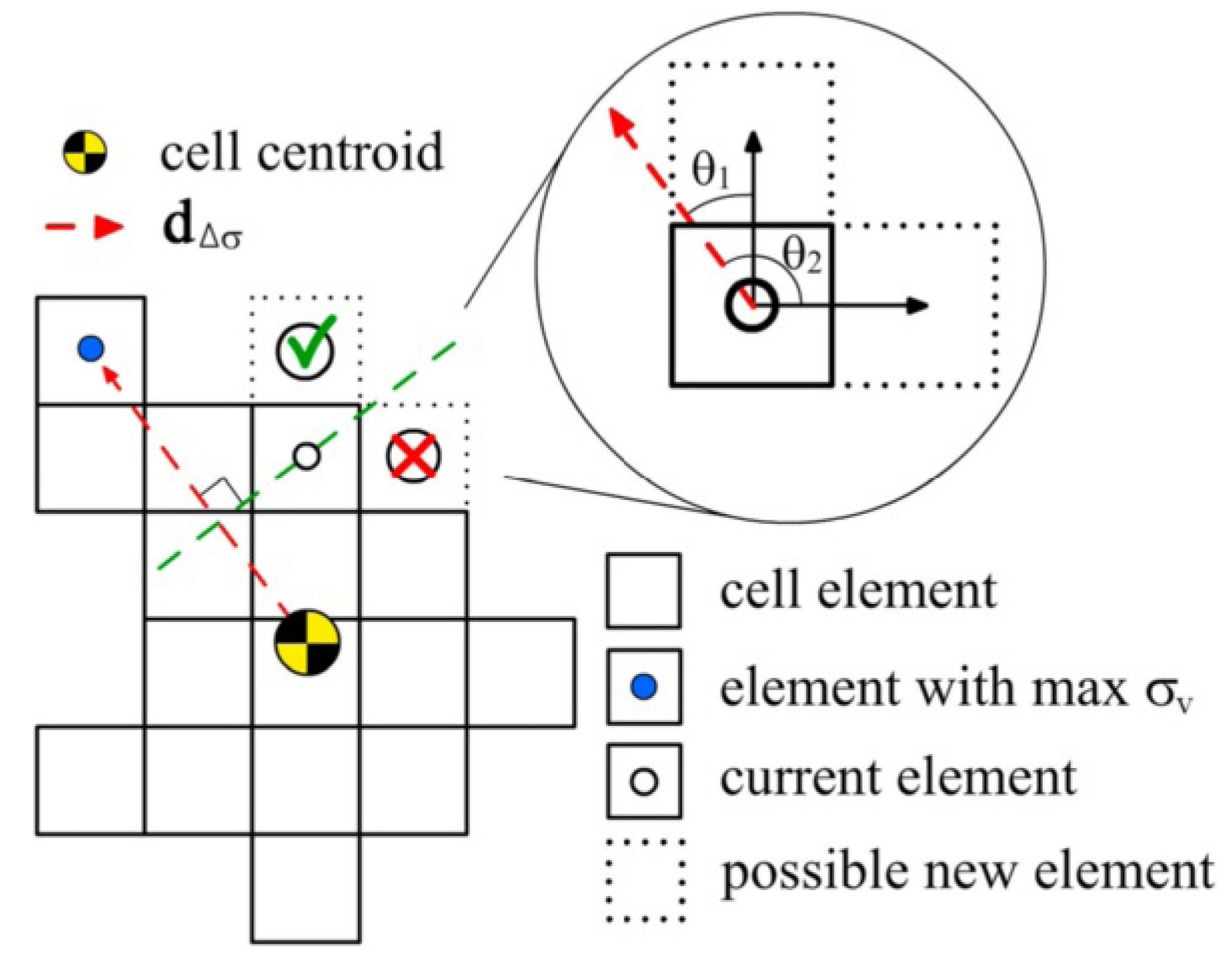
Schematic example of voxel addition process Voxel addition example taking only the stress direction and magnitude into account. When checking a specific voxel (current element), the volumetric stress that it bears (*σ*_v_) and the angle (*θ*) that its neighbours form with the direction of maximum stress (**d**_Δ*σ*_, red arrow) determine the probability of appearance (*p*_+_). In the illustration, the top voxel (currently part of the ECM) would have a higher probability than the right one of becoming cell since *θ*_1_ is lower than 90 degrees whereas *θ*_2_ is higher. Note that this is a simplified 2D scheme. In 3D, 6-connectivity is used to compute the voxel addition.

In this model, four different factors are considered to account for the mechanical, chemical and flow conditions surrounding the cell and driving cell migration. Namely these factors are: cell stress magnitude, maximum stress direction, chemical concentration at the ECM and flow direction. The volumetric cell stress (*σ*_v_) due to cell contraction is computed at each voxel following the previous *mechanosensing* model [22]. Here, the maximum stress direction (**d**_*Δσ*_) is defined as the direction in the cell body where the cell is exerting maximum stress. In other words, it is the direction joining the cell centroid (computed geometrically) with the element of maximum stress (Figure 4). The chemical concentration (*C*_c_) is a scalar field coming from the fluid chemical analysis, having each voxel an associated value. Similarly, **d**_f_ stores the flow direction corresponding to each voxel of the ECM. To define the addition/removal of voxels depending on the stimuli, these factors are introduced into the cell-dynamics or probability functions following the classical cumulative distribution [37]:

(5) $$p_{*}=p_{*}^{0}+p_{*}^{\max}\left(1-e^{-k_{*}^{0}\left(\lambda_{*}^{\sigma }F_{*}^{\sigma }+\lambda_{*}^{\Delta \sigma }F_{*}^{\Delta \sigma }+\lambda_{*}^{C}F^{C}+\lambda_{*}^{F}F_{*}^{F}\right)}\mathrm{dt}\right)$$

where * represents addition (+) or removal (−) of voxels. *p*^0^ and *p*^max^ are the minimum/maximum values bounding the probability. *k*^0^ is a temporal rate affecting all the factors and *dt* is the time step. In addition *λ*’s are sensitivity constants permitting to control the weight of each factor (*F*). All these parameters are adjusted to obtain cell speeds within a biological range. In addition, the values of these parameters are held constant during the simulation. Their values are listed in Table 2. On the other hand, *F*’s are variable parameters describing the environment conditions, different for each voxel and depending on the aforementioned stimuli. Each *F* ranges from 0 to 1 and they are described in the subsequent sections. A sensitivity analysis of the cell-dynamics functions was performed to study the global influence of each separate factor (Additional file 1).

The parameter representing the cell stress magnitude (*F*^*σ*^) measures the stress born in a specific voxel compared with the maximum possible cell stress (*σ*_max_) (eq.6), which value comes intrinsically from the *mechanosensing* model. The probabilities of adding/removing voxels, increase with the stress to reflect that cells embedded in stiffer substrates exert higher forces and move at faster speeds [2,5,8,38], This parameter also takes into account the voxel orientation. When adding a voxel, *θ* represents the angle between the direction of the possible new voxel (relative to the current voxel) and the direction of the voxel with maximum cell stress (Figure 4). In contrast, when removing a voxel, *θ* stands for the angle between the direction of maximum stress and the direction connecting the current voxel centroid with the cell centroid. Using this criterion, the probabilities of adding/removing voxels in the direction where the cell exerts maximum stress are higher/lower so the cell body tends to polarize, as suggested in experiments [10]. The alignment with stress is included in addition and separately with the parameter *F^Δσ^* in order to independently control the weights of the stress magnitude and stress gradient factors (eq.6):

(6) F+σ={σvσmaxcosθθ<π20π2≤θ}F−σ={0θ<π2σvσmax∣sinθ∣π2≤θ}F+Δσ={cosθθ<π20π2≤θ}F−Δσ={0θ<π2∣sinθ∣π2≤θ}

To further clarify this point, a simple 2D representation of the voxel addition process is shown in Figure 4. When checking a specific voxel of the cell surface (current), the corresponding value of stress and the position of its neighbours (possible new cell-voxels) are used to compute *p*_+_. In the illustration, the top voxel (which is currently part of the ECM) may become cell because *θ*_1_ is lower than 90° so $F_{+}^{\sigma }$ and $F_{+}^{\Delta \sigma }$ take a positive value depending on the stress and the alignment. On the other hand, the voxel on the right will not likely appear since *θ*_2_ is higher than 90° so $F_{+}^{\sigma }$ and $F_{+}^{\Delta \sigma }$ are 0 and hence $p_{+}=p_{+}^{0}$. Taking all this into account, the cell tends to migrate to stiffer zones of the ECM (higher cell stress) and in the direction of maximum stress.

It is well known that cells sense the ECM interstitial flow and respond to the concentration of a wide variety of chemical species [11-13,39]. To reflect this, both factors are included into the probability functions. The necessary inputs come from the fluid chemical analysis previously described. The parameter representing the chemical concentration (*F^C^*) compares the chemical gradient between adjacent voxels (*ΔC*) and it is normalized by the maximum value of concentration of a particular species (*C*_max_).

(7) F+C={ΔCCmaxΔC>00ΔC<0}F−C={∣ΔC∣CmaxΔC<00ΔC>0}

With this definition, the voxels tend to be added in the direction of maximum chemical concentration, appearing at a faster rate the more pronounced the gradient is. Similarly, the voxels tend to be removed more readily at the positions of lower concentration. In sum, the cell body advances in the direction of the chemical gradient. Obviously, in case of repellent species, *F^C^* could be easily reversed to account for opposite effects.

The dependence of cell migration on flow conditions have been recently investigated [13]. It was found that small populations of cells tend to migrate downstream and parallel to the flow direction. Actually, very high flow velocities acting on isolated cells or blocking of some specific receptors may reverse this response, although these effects are not considered here for simplicity. The flow parameter *F^F^* is then defined as:

(8) F+F={cosϕϕ<π20π2≤ϕ}F−F={0ϕ<π2∣sinϕ∣π2≤ϕ}

where *φ* establishes the alignment of the voxel with the flow direction array at a specific position. Therefore, *φ* is also calculated following the procedure shown in Figure 4, but using **d**_F_ instead of **d***_Δσ_*.

## Results and discussion

It has been shown that multiple combined factors drive cell migration through 3D ECMs, the properties of which influence the cell-matrix interactions and determine cell movements and orientation. This model focuses on three of these factors: fluid flow, chemistry and mechanical conditions. First, flow and chemical conditions of a real 3D microfluidic device [13] are simulated obtaining pressure distribution, chemical gradients and stream lines through a collagen ECM (porous matrix). Then, since the distance magnitudes that a single cell is able to migrate in a few hours (simulated time) are much shorter than the microdevice size, a central region of the gel is selected to compute the mechanical analysis.

Hence, this section is divided in three main parts. The first one summarizes the results from the microfluidic system simulation, showing the flow velocity field, the streamlines and the pressure gradient across the gel. The second part shows the effect of the ECM stiffness on the cell stress distribution and cell morphology. Finally, the results focus on cell migration, describing trajectories, speeds and directionality for different situations. Specifically, input factors (mechanics, flow or chemistry) are activated or deactivated in different combinations, thus altering the probability functions, and boundary conditions such as gradient directions are varied.

### Microfluidic simulation

**Figure 5:**
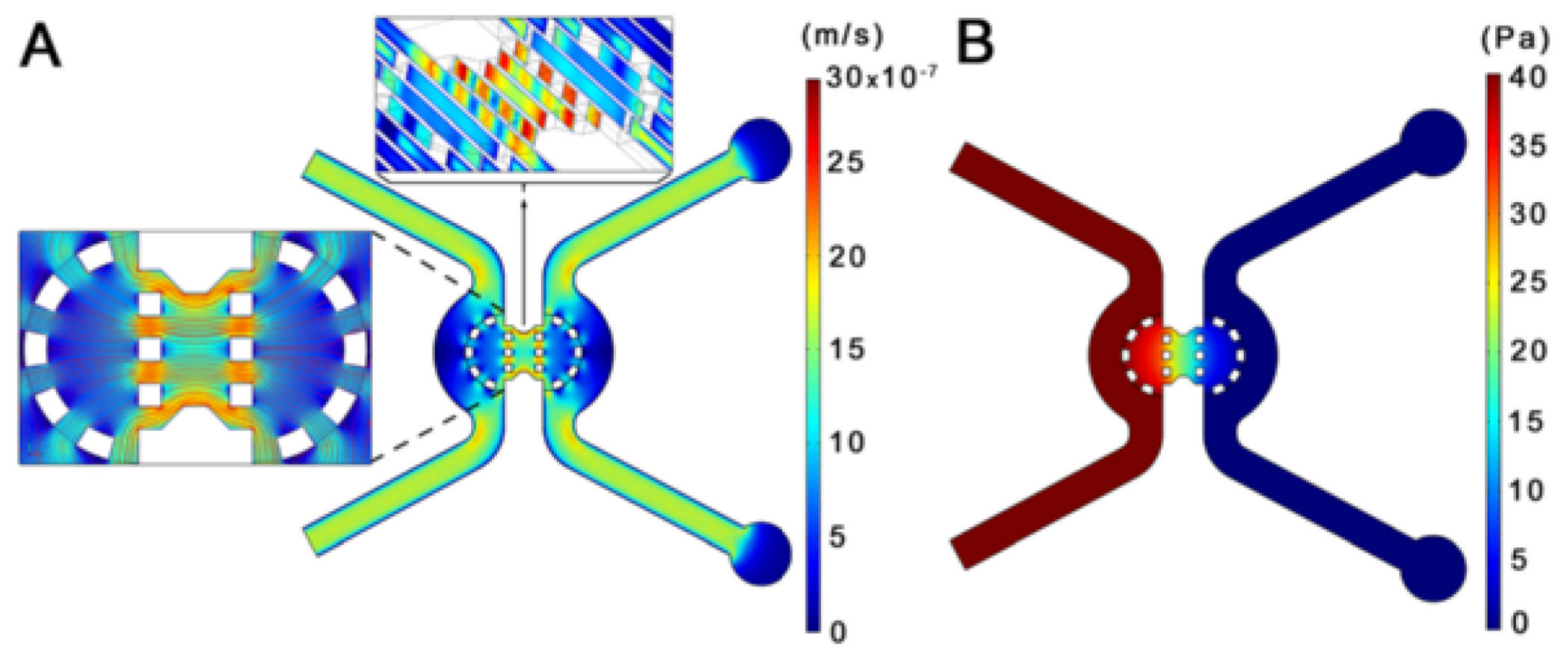
Fluid chemical analysis in a 3D microdevice **A)** The velocity field present higher values in the gel zone between micropilars, reaching a maximum of 2.96 μm/s. The streamlines in the central part are mostly parallel to the horizontal direction. **B)** The pressure drop across the microdevice shows a linear decrease through the gel and constant values at the inlet and outlet (40 and 0 Pa respectively). This analysis is computed (using COMSOL) once at the beginning of the simulation and its results are interpolated to a box-like voxelized mesh, where the mechanical analysis is performed and the cell migration is studied.

A full 3D microfluidic device is simulated with the conditions described in the FE analysis Methods section. The fluid passes by two input channels and flows through a porous medium (collagen gel) transporting a certain diluted specie, and achieving its peak speed (2.96 μm/s) at the central zone of the gel, between the micropilars, where the cross section is smaller (Figure 5A). The velocity field matches quantitatively the results obtained both computational and experimentally by Polacheck et al. [13], which found a maximum speed of about 3 μm/s. The pressure drop presents a linear decrease through the gel and constant values at the inlet (40 Pa) and outlet (0 Pa) (Figure 5B). Similarly, the chemical concentration at the gel decreases linearly from a normalized value of 1 mol/m3 at the inlet, to 0 mol/m^3^ at the outlet (not shown). which allows testing the migration model using this additional factor. Future development of the model could incorporate the transport of different species or autocrine gradients produced by the cell, although they were not considered in the present simulation.

### Effects of ECM stiffness

To test the direct effects of ECM stiffness on cell morphology and stress distribution, a box-like domain (300 × 300 × 120 μm) with constrained displacements at the boundaries (far enough from the cell to avoid influencing the *mechanosensing* process described in the methods section) and different ECM stiffness conditions was used. Up to 10 simulations were performed for each set of conditions with mechanical stimulus acting alone (flow and chemical inputs deactivated). These simulations presented some differences due to the stochastic nature of the model, but overall all the results were consistent. For clarity, only one simulation of each set of conditions is presented. For all the cases shown here, the cell was assumed to have an initially spherical shape of ~30 μm of diameter and started the simulation in the domain centre (Figure 2). Time simulated was 500 min (100 steps) which is in the usual range of cell migration experiments [8,13]. Model parameters were adjusted to predict speeds similar to migrating fibroblasts observed in experiments [5,8,38,40,41].

**Figure 6:**
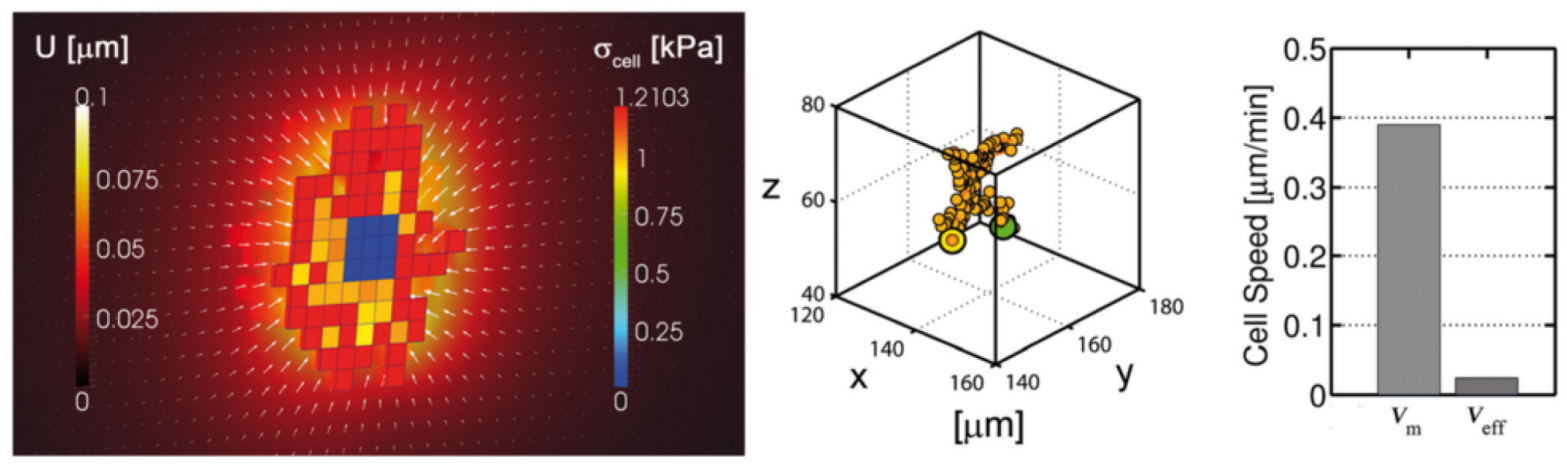
Cell response for homogeneous ECMs. Volumetric cell stress, ECM displacement (left), 3D trajectory (middle) and migration speeds (right) for a case with homogeneous stiffness (50 kPa) Left plot shows a cut of the cell body. Cell stress is distributed homogeneously (red cell-voxels) along the cell surface and slightly decreases in the cytoplasm zone. Note that the plot only represents the active stress exerted by the cell elements and not the stress transmitted to the ECM or the nucleus. The nucleus is considered a passive material, thus appearing in blue. ECM displacements are distributed homogeneously, pointing radially to the cell centroid (left legend and white arrows). Middle plot shows cell migration trajectory. Having no guidance, cell moves randomly, which is reflected in the low effective speed.

First, the cell is embedded in a homogeneous ECM with constant elastic modulus of 50 kPa. This value is larger than the modulus corresponding with the 2 mg/ml collagen gel used in the simulated microdevice [13]. Nevertheless, we used this higher value to show the effects of stress saturation with stiffness, as we explain later. With no stiffness anisotropy, the ECM displacements are homogeneously distributed, pointing radially to the cell centroid. Similarly, the cell stress is mostly homogeneous, with higher values at the cortex zone (~1.2 kPa) and slightly lower ones in the cytoplasm (Figure 6, left). These values are in the order of magnitude of cell stresses found in experiments [32-35]. In addition, considering the surface of each voxel face (9 μm^2^), the magnitude of cell forces would be in the correct range (up to few hundreds of nN) of experimental data [42-44]. Note how the nucleus (assumed passive), is being stretched by the surrounding contracting elements. With such homogeneity, the chance of adding/removing elements at the cell surface is similar in all directions (see methods) and consequently, the cell migrates in a random fashion (Figure 6, middle). Also note that the migration speed depends on the ECM stiffness through the probability functions since higher stiffness lead to higher cell stress (until saturation) and thus to higher migration speeds. In this case, results show ~0.4 μm/min of mean speed and ~0.024 μm/min of effective speed (Figure 6, right). Mean speed is calculated as the average cell speed at each step, whereas the effective speed takes into account only the initial and final cell location at a certain time. Low effective speed reflects high randomness.

**Figure 7:**
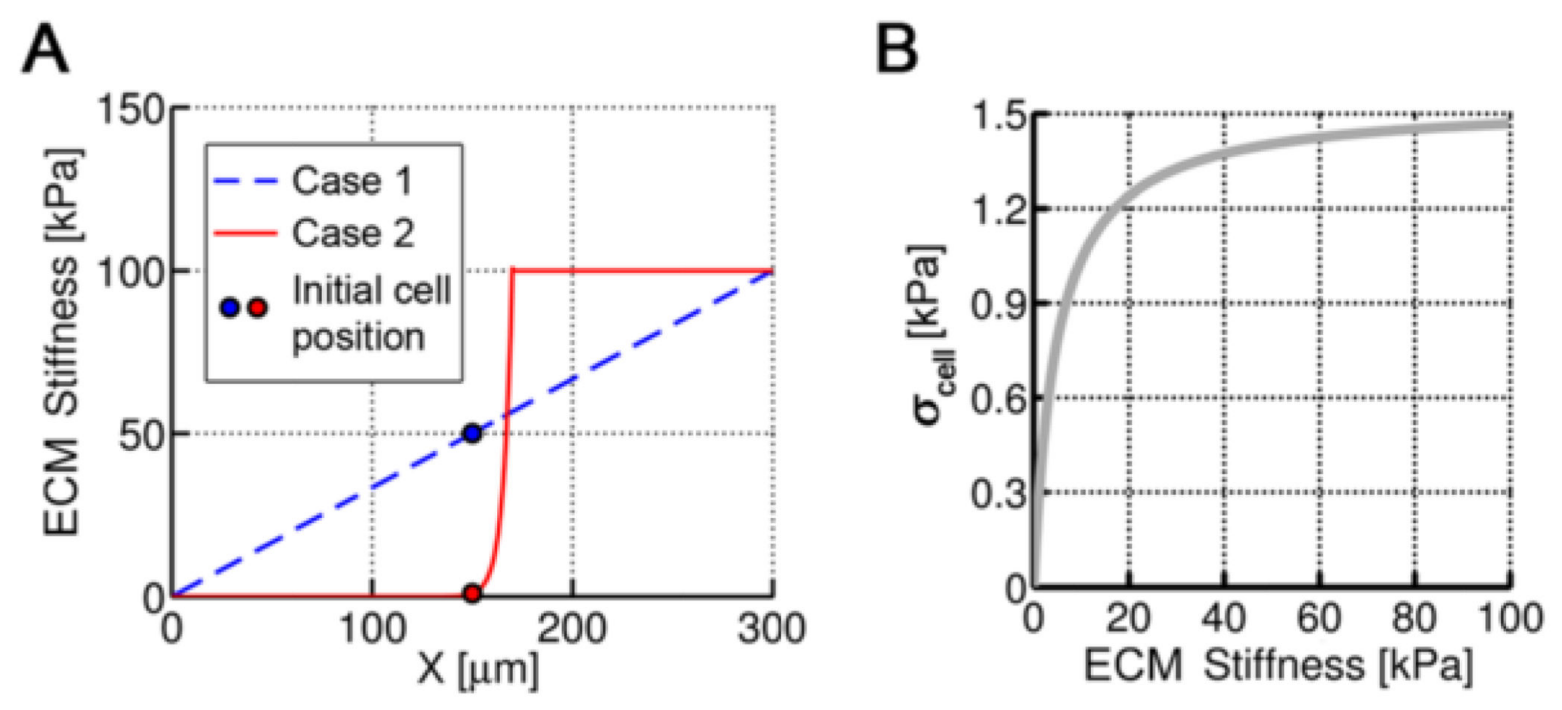
ECM stiffness gradients and theoretical cell stress **A)** Two different cases are simulated. The ECM stiffness varies linearly with x-coordinate in the first case and exponentially in the second one. The cell starts the simulations at the same location but surrounded by different compliant ECM depending on the gradient type. **B)** Cell stress depending on ECM stiffness. Note that this curve corresponds with the theoretical solution of the mechano-sensing model in one direction, that is, the stress of one single voxel completely surrounded by an elastic ECM of a specific stiffness.

**Figure 8:**
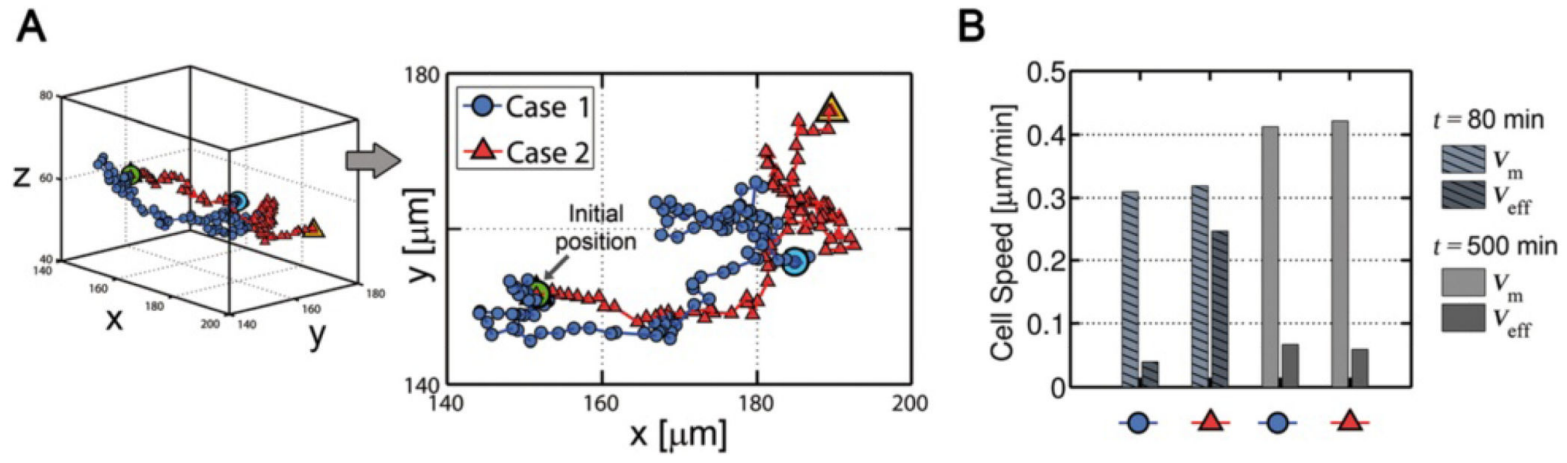
Migration trajectories and computed speeds **A)** 3D and x-y projected trajectories for: case 1 - linear stiffness gradient, case 2 - exponential stiffness gradient. Initial position is the same for both cases. Light blue circle and orange triangle show the final location of the cell centroid for cases 1 and 2 respectively. **B)** Cell migration speeds at different times of simulation. Legend in A is used to represent the cases in the x-axis of B. Mean speed is calculated as the average cell speed at each step, whereas the effective speed takes into account only the initial and final cell location at a certain time.

Secondly, two cases with different stiffness conditions are simulated. In case 1, the elastic modulus of the ECM increases linearly with x-coordinate, whereas in case 2, the increase is exponential (Figure 7A). The cell centroid at each step is tracked and the 3D and x-y projected trajectories are shown in Figure 8A. Overall, in both cases, cell migration pathways were random with a higher net advance in the direction of the gradient stiffness (x-direction). However, cell response was different, moving slightly faster but much more directed in case 2, especially during the first steps. In this case, the stiffness variation (and thus, cell stress) between the front and the back part was very pronounced. According with the probability functions, this corresponds with much higher probability of voxel appearance in + x-direction and of voxel removal in −x-direction, resulting in fast forward advance. This was reflected on the mean and effective speeds of cell migration (Figure 8B). For short times, the mean speeds were similar in both cases (~0.3 μm/min), but the effective speed was much higher in case 2 (0.25 μm/min compared with 0.04 μm/min in case 1), as expected from the trajectory analysis.

**Figure 9:**
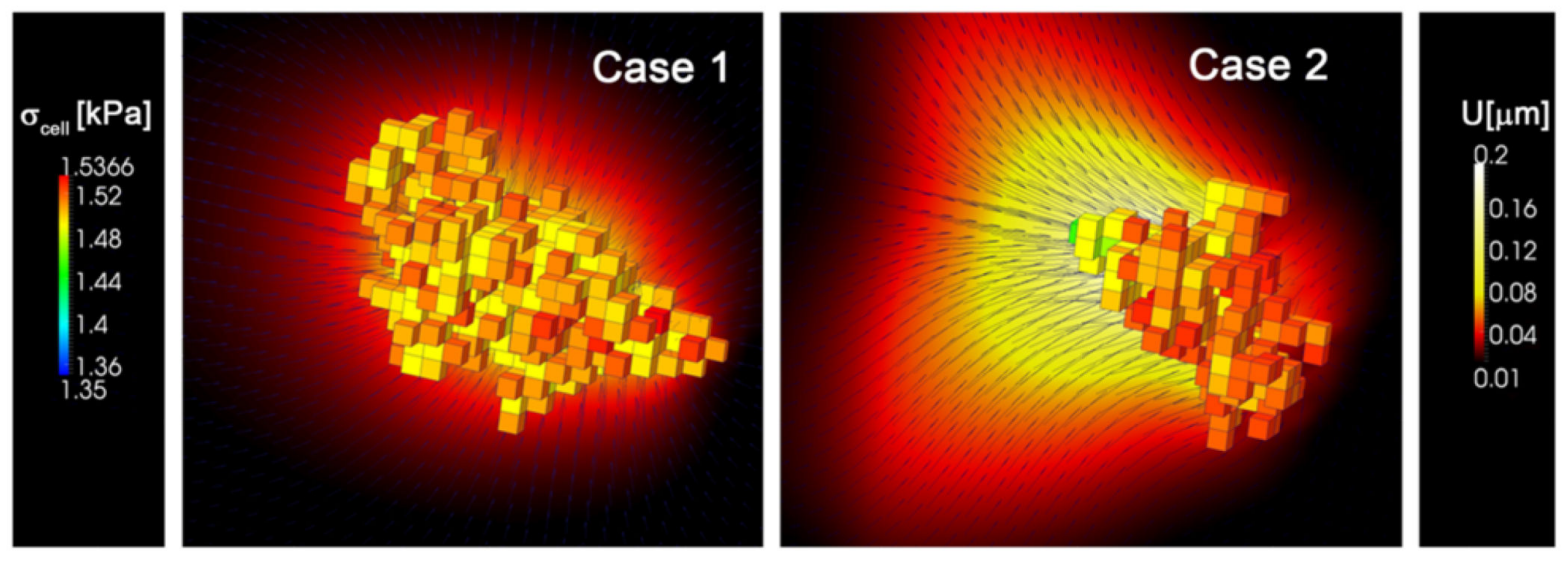
Cell stress and ECM displacements Cell stress (coloured voxels) and ECM displacements (black arrows and cut plane) at *t* = 80 min for case 1 (linear stiffness gradient in x-direction) and case 2 (exponential stiffness gradient in x-direction).

However, for long-term, both case 1 and 2 presented similar mean (~0.42 μm/min) and effective (~0.06 μm/min) speeds, and the trajectories were mostly random. This is due to cell stress dependence on ECM stiffness. According to the *mechanosensing* model, cell stress increases with ECM stiffness, swiftly for compliant substrates but saturating for higher rigidities (Figure 7B). As stated before, pronounced differences between front and rear stress would cause fast and straight movements, whereas small differences would lead to random-like migration. In case 1, cell moved between stiffness of 45–65 kPa, always close to the saturation zone, which explains its non-directional motion. On the other hand, in case 2 the cell started in a compliant zone (1 kPa), but quickly found much stiffer surroundings (100 kPa) which highly increased cell stress, decreasing back and rear differences and thus producing stochastic migration. Figure 9 shows the stress distribution for both cases at *t* = 80 min which is approximately the time at which the cell arrived to a very stiffer zone, reaching force saturation and thus migrating more randomly. In case 1, cell stress is homogeneously distributed, although the voxels with higher stress corresponded with surface (cortex) elements preferentially oriented in + x-direction. Cell shape is mainly regular but generally polarized with the gradient direction, and the ECM displacements point radially to the cell centroid. In case 2, however, there exist a clear gradient of cell stress following the ECM stiffness. The cell shown in Figure 9 presents a shape which is broader at the front, exerting higher stress, and very thin at the rear. Nevertheless, due to the pronounced stiffness gradient, displacements are much higher at the rear and the ECM is mainly stretched in the x-direction.

**Figure 10:**
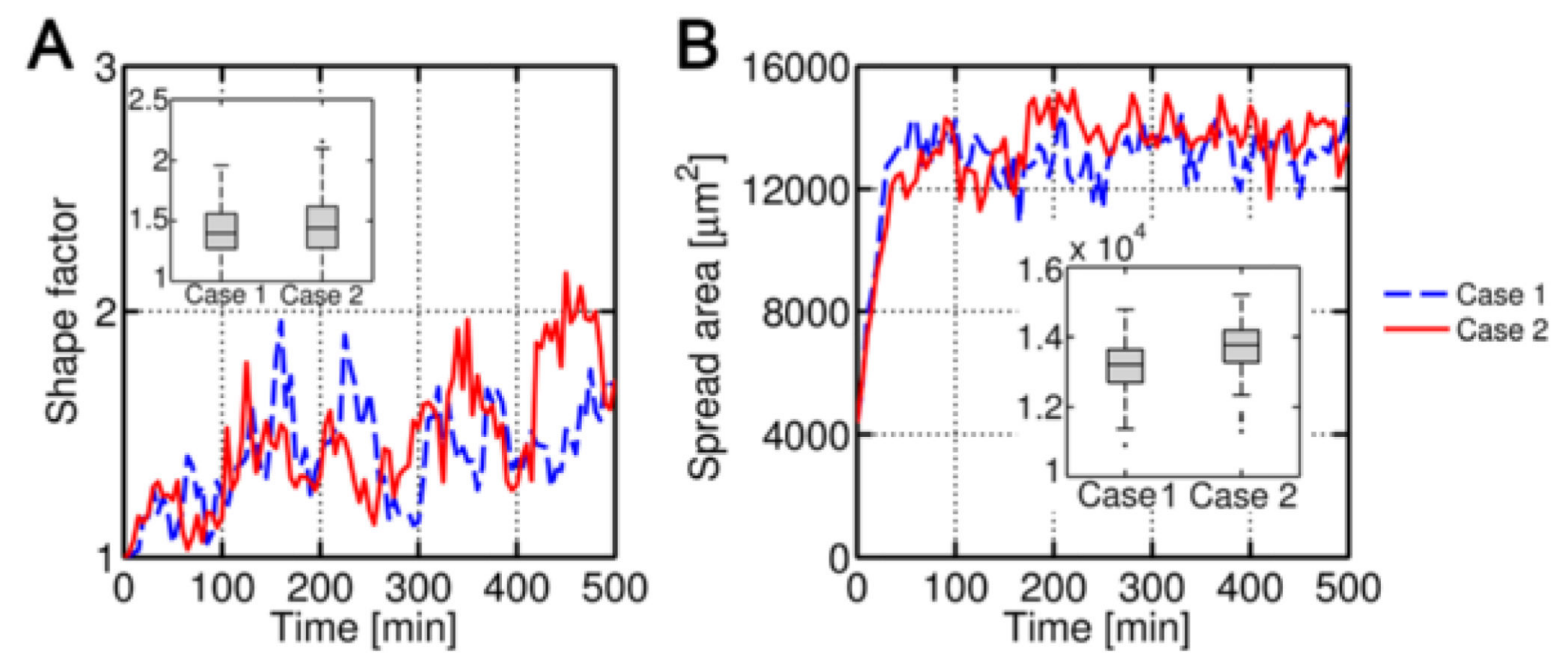
Cell shape factor and spread area **A)** Cell aspect ratio and spread area **B)** for case 1 (linear stiffness gradient in x-direction) and case 2 (exponential stiffness gradient in x-direction) **B)**.

Overall, cell aspect ratio or shape factor (major axis divided by minor axis) (Figure 10A) was similar for both cases, as well as the spreading area (Figure 10B), presenting case 2 slightly higher values. This likely happens for the same reasons explained above. The probability functions tend to saturate at high stresses and hence the voxel appearing/disappearing probability is high in all directions. Therefore the aspect ratio is noisy and relatively low, from roundish-like shapes to somewhat elongated (2:1) cells.

#### ECM degradation

The matrix metalloproteinases (MMPs) are a family of ECM degrading enzymes which play a major role on cell behaviors such as migration, differentiation or angiogenesis. In fact, localized matrix degradation is thought to contribute to cellular invasiveness in physiological and pathological situations [45]. This degradation modifies the morphology and mechanical properties of the ECM, therefore affecting the cell behavior. Computational modeling of such a complex phenomenon requires specific and focused research [29]. Nevertheless, the possibility of ECM degradation was added into the codes for possible future development.

**Figure 11:**
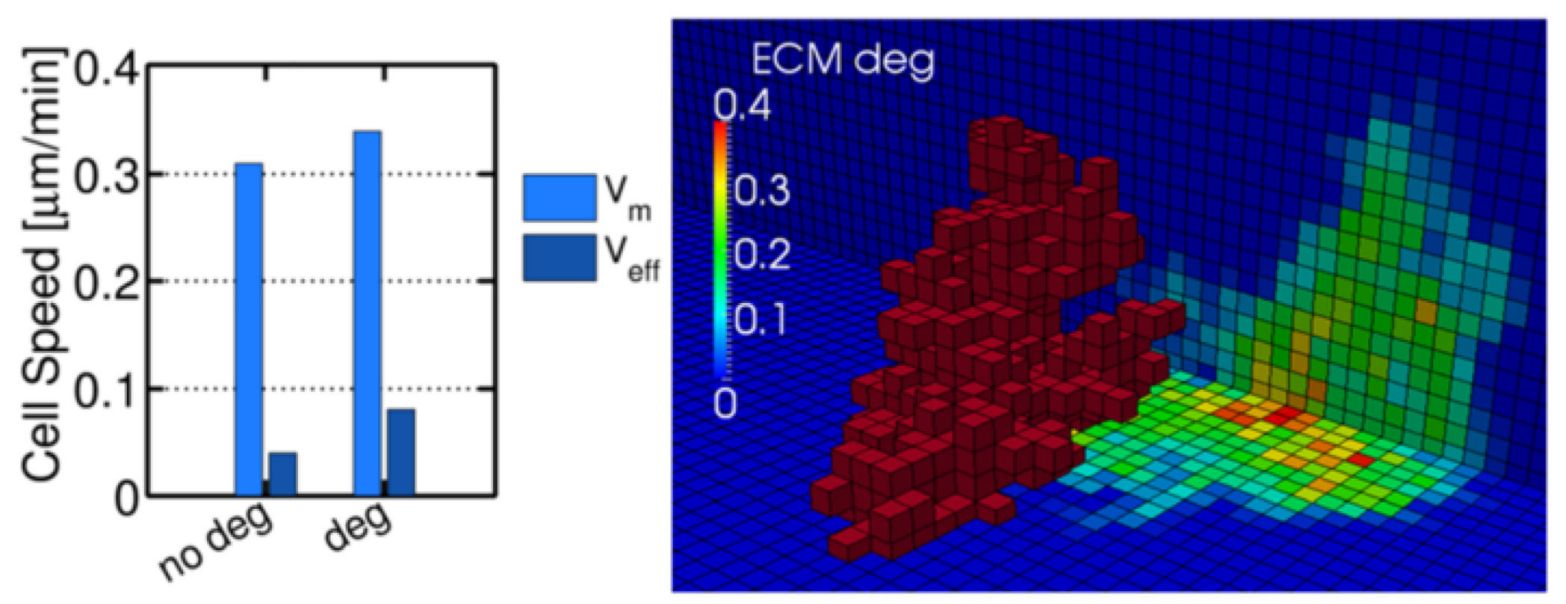
Cell speeds and ECM degradation **A)** Cell speeds and matrix degradation **B)** for case 1 (linear stiffness gradient in x-direction) at *t* = 80 minutes. Cell speed slightly increases while the cell leaves a degraded path at the trailing edge. Red voxels represent the cell, whereas coloured background shows the percentage of ECM degradation.

As a first approximation, a very simple rule was incorporated: whenever an ECM-voxel (i) is in contact with the cell perimeter it becomes degraded, losing a certain percentage (*d*) of its original Young’s modulus $\left(E_{\mathrm{ECM}}^{i}=E_{0}(1-d)\right)$. To test the effect of such simplification, case 1 (linear stiffness gradient in x-direction) was computed again activating ECM degradation (using *d* = 0.01). Results after 80 minutes of simulated time show that both the effective and mean speeds increase when the ECM is degraded (Figure 11 left). The reason is that the degradation of the ECM mechanical properties (lower E) decreases the probabilities of adding cell elements at the trailing edge. Thus, the cell tends to migrate faster leaving a degraded path on its way (Figure 11 right).

Further development of a degradation model might be interesting in the future, although the degradation option was deactivated in the main simulations for simplicity, to isolate the effects of the rest of phenomena.

### Migration

**Figure 12:**
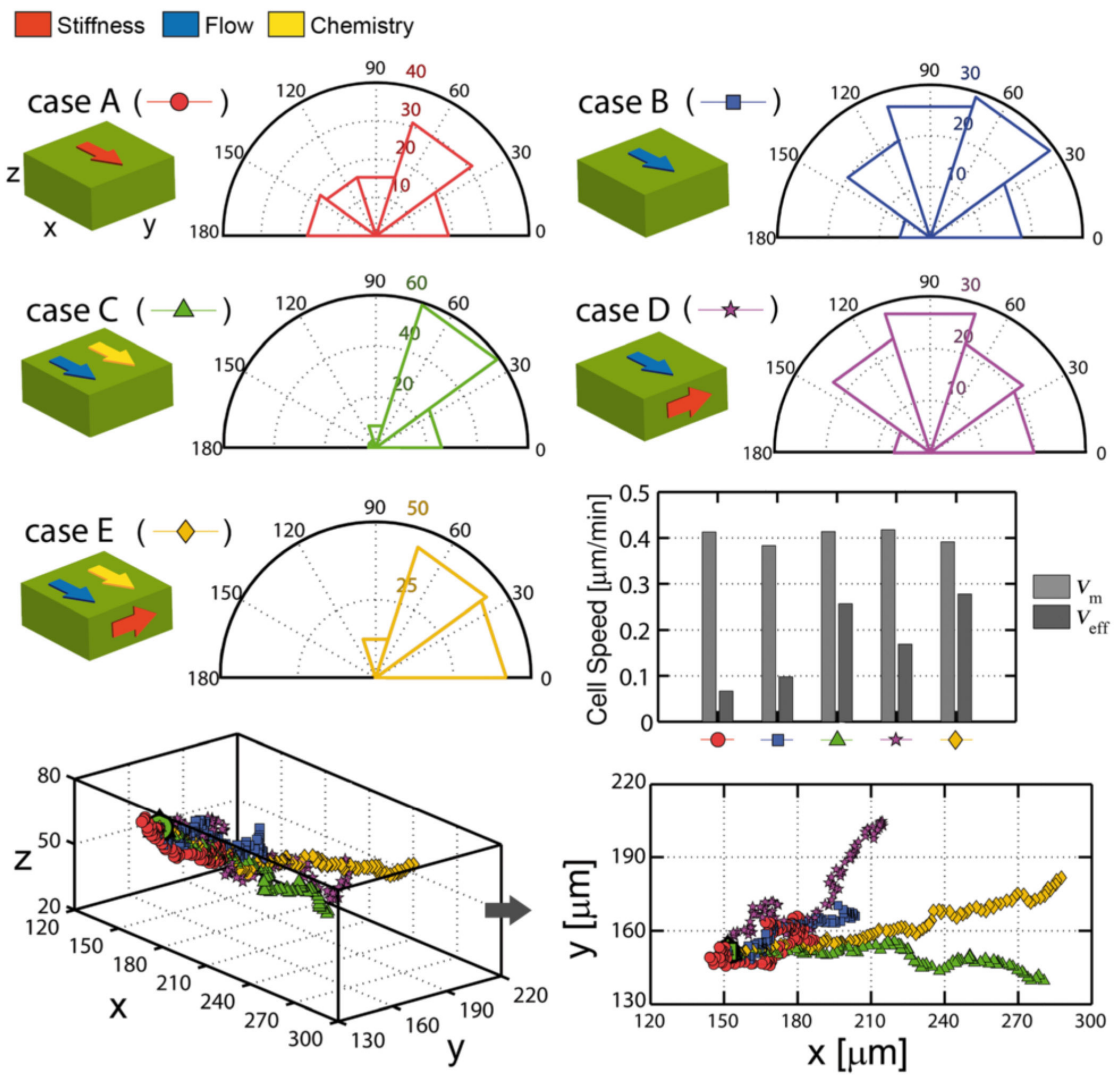
Cell migration under different environmental conditions Mechanical, flow and chemical inputs are activated/deactivated in different combinations and gradient directions. Case A: only the mechanical input is activated, applying a linear stiffness gradient (same as case 1 in previous section) on the x-direction. Case B: flow acts in x-direction. Case C: flow and a chemical gradient are both applied in x-direction. Case D: flow is applied in x-direction and a stiffness gradient in y-direction. Case E: flow and a chemical gradient are applied in x-direction and a stiffness gradient in y-direction. Green box represents the gel and coloured arrows the gradient directions. Migration directionality was determined as the angle of each turn in the track relative to the x-direction. Coloured numbers represent the count of turns at each simulation.

To study the resulting patterns depending on input environmental factors by activating/deactivating mechanics, flow or chemistry, and using different combinations of gradient directions, 500 min (100 steps) of cell migration were simulated. Five specific cases were distinguished (Figure 12): (A) only mechanical inputs activated, applying a linear stiffness gradient (same as case 1 in previous section) on the x-direction, (B) migration is only driven by fluid flow in x-direction, (C) flow and a chemical gradient are both applied in x-direction, (D) flow is applied in x-direction whereas there is a stiffness gradient in y-direction, (E) flow and a chemical gradient are applied in x-direction and a stiffness gradient acts in y-direction.

Down panel of Figure 12 shows the 3D trajectories and the x-y projection. Mean and effective velocities at the end of simulation are plotted for each condition. Although the mean or averaged speed (*V*_m_) was similar for all the cases (~0.4 μm/min), the effective speed (*V*_eff_) was strongly influenced by the boundary conditions. For each case, the directionality of the migration as the angle of each turn in the track relative to the x-direction was determined. Results reflect the sensitivity of the model when applying single or combined factors. Stiffness or flow gradients acting alone (cases A,B), produced more random migration with ~40% of backward movements, which is reflected on effective speeds under 0.1 μm/min. Introducing a second factor on the x-direction (case C), even when another gradient was acting in the y-direction (case E), substantially decreased the randomness. In these cases, only ~10% of the turns went away from the “correct” path, overall achieving effective speeds of ~0.25 μm/min. Interestingly in case D, where the gradients are applied in x and y-directions, the effective speed (~0.16 μm/min) was greater than in cases A or B, probably due to the fact that random deviations were combined with either the direction of the stiffness or the flow gradient.

### Modeling a porous ECM

So far, all the simulations have considered a continuum matrix through which the cell is able to migrate, completely neglecting morphology or geometrical effects of the ECM. In this section, a porous mesh is simulated to compute cell migration through the matrix pores.

**Figure 13:**
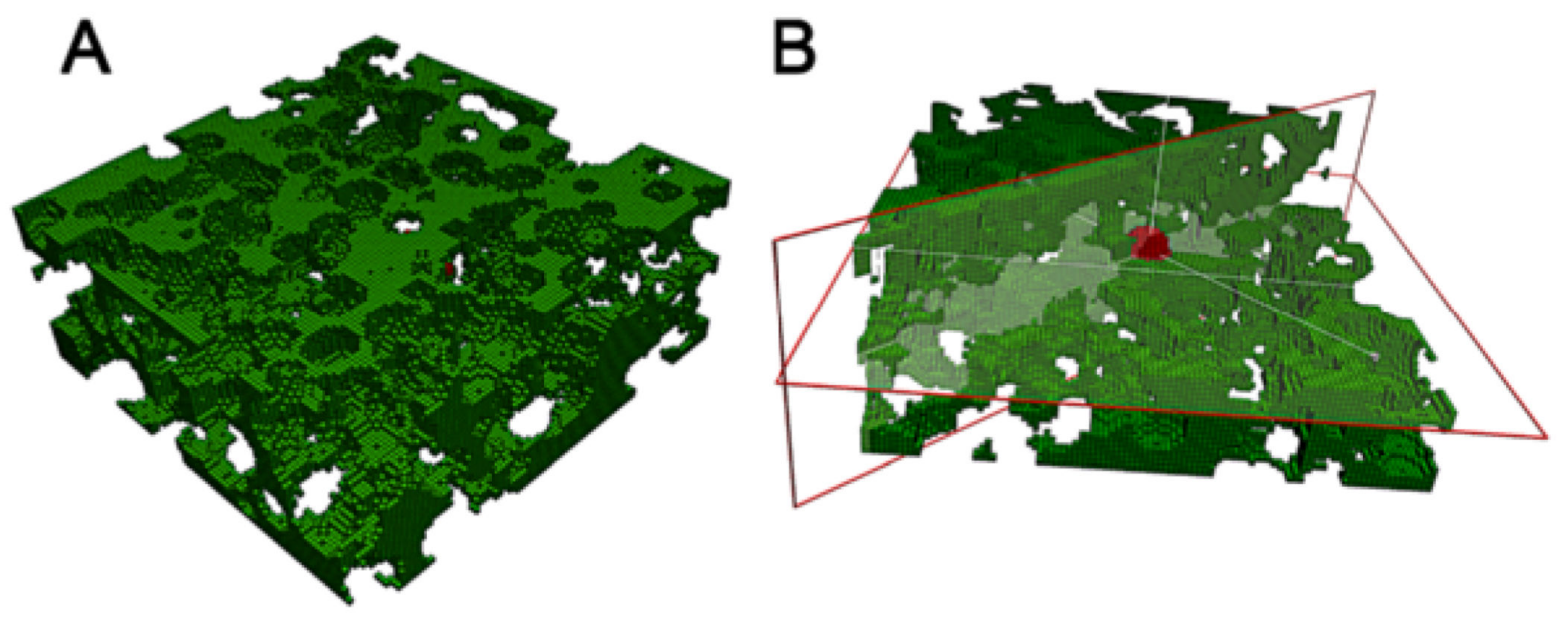
Example of a porous ECM voxel-mesh **A)** Mesh of porosity ~0.9 and average pore size ~20 μm. **B)**. Domain cut using horizontal and diagonal planes showing cell’s initial position.

**Figure 14:**
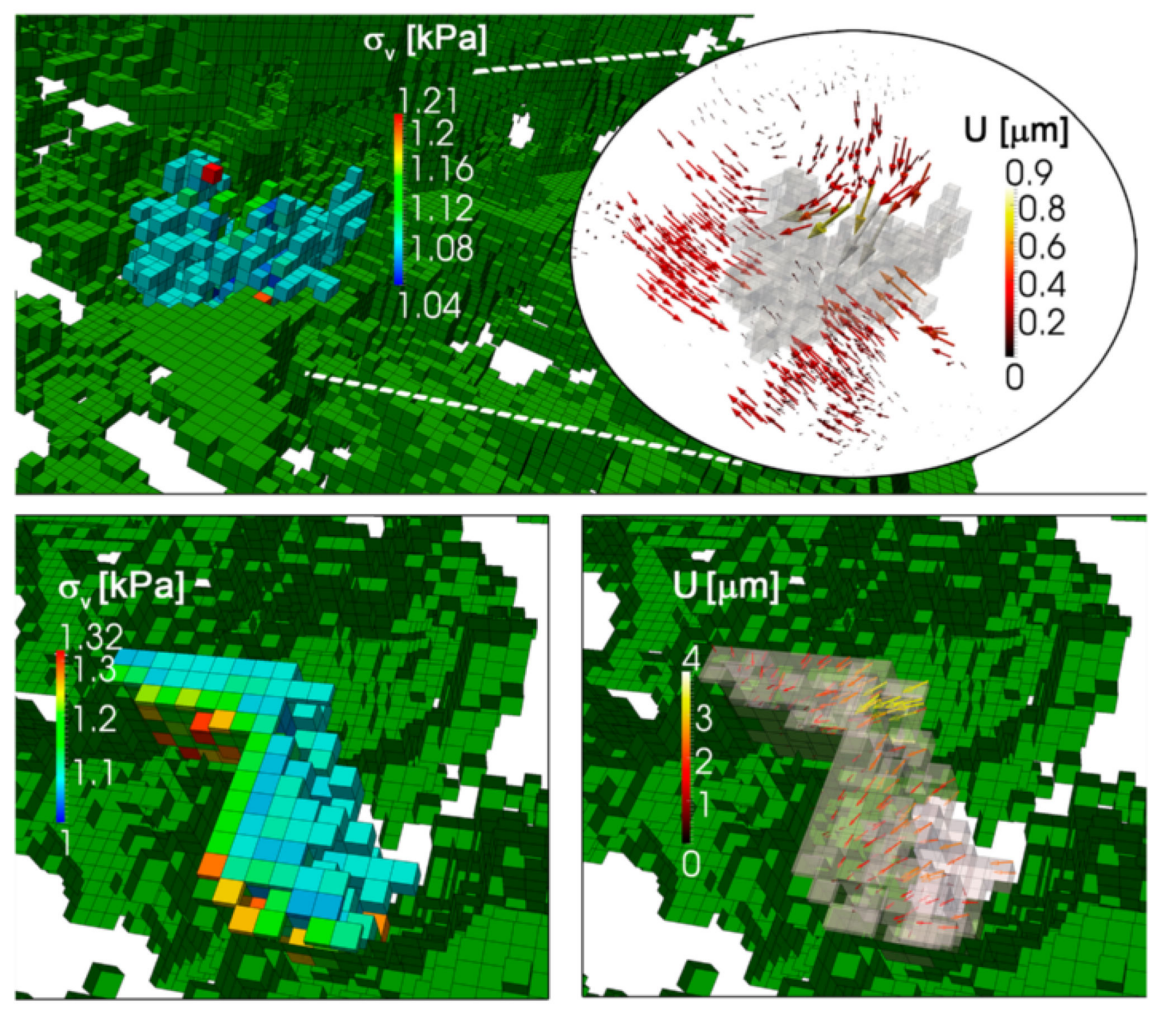
Cell stress and displacements in a porous ECM Top panel shows cell stress and ECM displacements (significantly higher than those in a continuum ECM). Bottom left panel shows cell stress and the cell body adhered to the pore surface (where it develops higher stress). Cell body contracts toward the pore surface (bottom right panel), with high displacements at the free side.

The domain size is the same as used in previous simulations (300 × 300 × 120 μm with voxels of 3 μm) but the mesh is performed randomnly obtaining a porosity of ~0.9 and average pore size ~20 μm (Figure 13A). This pore size is large, especially for physiologic matrices, however, since we are not introducing hindrance or other phenomena related with the cell advance through little pores, a bigger pore size is more adequate to study morphological changes of the cell body. The cell is initially placed at the domain center (note that cell’s volume is taken into account when building the mesh) (Figure 13B). The ECM is still considered as linear elastic for simplicity with homogeneous Young’s modulus of 5 kPa, and the cell behavior follows the *mechanosensing* model. In addition, the flow field in x-direction is interpolated from the microfluidic simulation. The observed cell behavior was similar to that found in previous simulations using continuum ECM’s, presenting, however, some peculiarities. Developed stress was similar to previous cases (~1–1.3 kPa) although ECM displacements were significantly higher (up to 0.9 μm) due to the pores (Figure 14). Interestingly, the cell tends to adhere to the pore surface, where the stiffness (and therefore the stress) is higher (Figure 14 bottom left). Moreover, the cell contracts its body toward that surface, presenting high displacements at the non-adhered voxels (Figure 14, bottom right).

**Figure 15:**
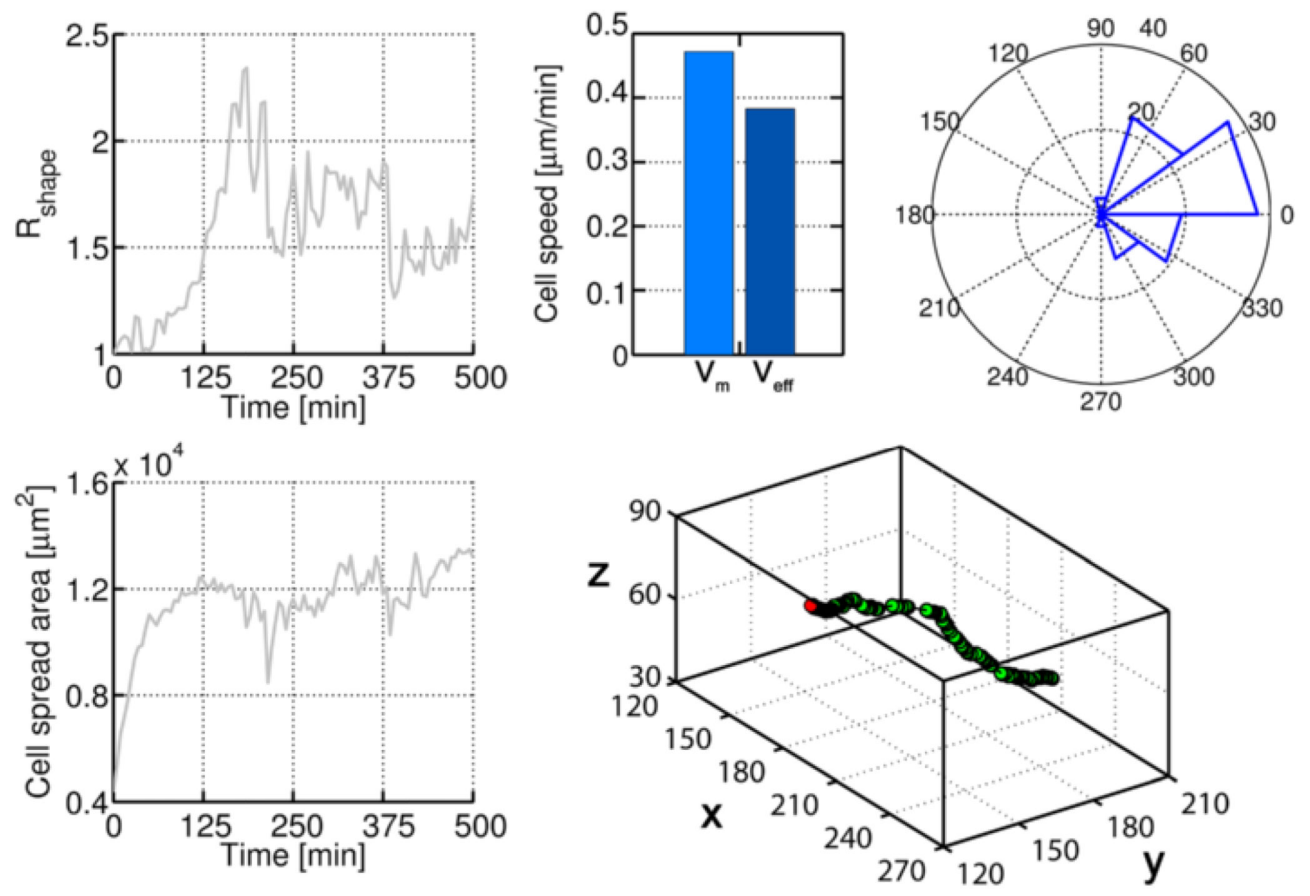
Cell response in a porous ECM Left plots show the cell shape factor and spreading area. Noise is caused by the irregular ECM geometry. Mean and effective speeds are similar, suggesting a directional migration, as confirmed by the trajectory and the angle distribution with respect to x-direction (right plots).

Mean and effective speeds were similar and high (above 0.35 μm/min), indicating a directional migration. In fact, both the trajectory and the angle distribution confirm that the cell moved mainly in x-direction, adhering to the pore surfaces but following the flow lines (Figure 15, right plots). Cell shape factor and spreading area present noisy behaviors due to the irregular ECM geometry, although the values are similar to those obtained in a continuum domain.

## Discussion

In this work, a phenomenological probabilistic voxel FE model for single cell migration in 3D has been described. Through a set of probability functions and combining different software, the model is able to compute cell migration taking into account different environmental factors evaluated at the cell surface such as mechanical properties of the ECM, chemical gradients, flow and boundary conditions, capturing important migration-related features such as cell speed, cell stress, ECM-displacements, spread area, cell aspect ratio etc. To study the fluid-chemical environment, a full 3D microfluidic device whose geometry and conditions were taken from a recent experiment [13] is simulated, in which the fluid passes by the input channels and flows through a porous medium. On the other hand, to analyze the mechanical environment, the mechanical equilibrium is solved by using a specific *mechanosensing* model. The macroscopic behavior of the cell emerges naturally from the definition of probabilities at each voxel (based on the conditions at the macro-scale), allowing the study at the micro and cell scales.

Overall, the model predicts cell migration toward stiffer zones of the ECM [5-8], downstream and parallel to the flow [13,39] and oriented with chemical gradients [11,12]. The parameters of the dynamic functions were adjusted to obtain migration speeds in the range 0–1 μm/min [5,8,38,40,41] and cell stresses of the order of few kPa as reported experimentally [32-35]. In addition, the effects of combined factors were investigated, confirming that the model responds accordingly in random but controlled fashion.

This approach joins together features from different kind of existing migration models. For instance, similarly to the force-based dynamic approaches, the mechanical equilibrium is locally established taking into account the cell contraction depending on ECM conditions following a *mechanosensing* model [22]. Note that although this approximation is sensitive to external loads (e.g. hydrostatic pressure or ECM pre-strains), only stress and strain caused by cell contraction are taken into account. Additionally, a 3D lattice is used, like in Monte Carlo studies, which usually permits faster simulations at the expense of quantitative results. Nevertheless, since the cell body is discretized with voxels, this handicap is skipped and the model is able to qualitatively and quantitatively study different aspects of cell migration. Obviously, this simplification implies other disadvantages such as the accuracy loss at the cell surface. In fact, it is important to remark the commitment between voxel and cell sizes. The number of voxel elements must be large enough to represent the cell perimeter but small enough to maintain a reasonable computational cost. The expected cell speed should also be taken into account. For instance, to simulate the migration of a slow cell, you the global size of the ECM could be decreased, and smaller elements can be used to increase the accuracy. Hence, in terms of computational cost, the best case would be a large and slow cell, and the worst a fast small cell (e.g. a bacterium). Unfortunately, a mathematical law to define the optimal voxel-size does not exist, although we found that one tenth of the global cell size was overall a good choice.

Finally this approach is based on probabilities. However, unlike purely stochastic models, ECM properties or cell stress can be included to drive migration. In fact, this first approach focuses on fluid direction, chemical gradients and mechanical cues as the main inputs driving cell migration through the probability functions. It is worth mentioning that the initial cell shape (assumed spherical at *t* = 0), would only affect the first migration steps. For instance, an initially elongated or polarized cell would steadily reorient according to the external inputs due to the probability functions, and therefore the general trend would be maintained. These tunable functions allow controling the relative weight of each input parameter (by varying the corresponding *λ*’s), as well as including new factors that affect cell migration. For instance, some experiments [13,39] suggest that cells polarize with the interstitial flow direction and migrate downstream due to a flow-induced gradient of an autocrine chemotactic signal that is detected by specific chemokine receptors. When those receptors are blocked or when the cell population grows (thus disrupting the signalling processes), the migration trend is reversed. This effect could be easily introduced in the model by simply switching the values of *F^F^* or including a signalling function regulating that specific parameter. Also, the model predicts increasing speed migration (higher probabilities) with ECM stiffness, not considering hindrance or drag effects that may appear in dense ECMs. To account for the biphasic behavior of cell speed versus ECM stiffness, as found in experiments and used in previous models [21,22,40,46,47], *F^σ^* could be modified so that the probability of adding/removing voxels decreased as a function of drag (*σ*_v_/(*σ*_max_*f*(*drag*))), or a specific *F*^drag^ with negative values could be defined.

Adding new input factors or enhancing current assumptions is thus possible and easy, although increasing complexity may complicate the interpretation of the results. Nevertheless, with the activation/deactivation of input factors, the model serves as a suitable platform for investigating a wide variety of migration-related phenomena. In fact, in a future development, it will be possible to deep further into some important aspects which are now oversimplified. For instance, ECM degradation could be easily included in the model to study differences between proteolytic and non-proteolytic migration. Additionaly, the ECM architecture could be further explored, studying the effects of porosity and pore size, including features of contact guidance or even reconstructing the geometry from real images. Furthermore, in this kind of environments, blebbing migration usually plays an important role as an alternative mode of migration [48]. Although the current model is based on the *mechanosensing* assumption (which implies cell-matrix adhesions) and internal pressure driving independent cell protrusions could be also incorporated. Another simplification is the assumption of a constant difference of maximum stress between the cortex and the cytoplasm. However, the complex reality could be better represented by making the maximum stress magnitude dependent on myosin activation or protein concentration along the different cell parts. Similarly, the stiffness of active cell components (*K*_act_) could rely on actin polymerization and cytoskeletal reorganization. These and other phenomena could be incorporated to better reflect the dynamics of cell migration.

Nevertheless, it is important to bear in mind the main handicap when working at different scales (microdevice vs. gel vs. cell), which is the computational cost. To solve this, different FE software (COMSOL Multiphysics) including a specific microfluidics module is used, and the steady-state solution of the fluid-chemical problem is computed. Then, this solution is interpolated into a finer mesh of the central part of the porous gel, where the mechanical analysis and cell migration are computed. Since the model simulates single cell motility, the cell volume does not affect the macro-scale results of the fluid-chemical simulation, and thus it can be neglected permitting considering the stream lines and chemical gradient constant during simulation. In spite of this assumption, the scripts require up to 30 GB of RAM memory, too high for a common personal computer. Furthermore, in case of extending the model to compute collective cell migration, the mentioned simplification would not be valid, making thus necessary a new approach and considerably increasing the computational cost. With all this, another limitation of the current model is the extended use of commercial software (ABAQUS, MATLAB, COMSOL) which restricts the sharing possibilities, although it is intended to remove this dependence in the near future by creating specific hand-coded routines.

## Conclusions

In sum, this work establishes a methodology for testing and designing new experiments; being in particular useful for simulating ongoing microfluidic systems and the study of several basic biological functions such as cell migration, angiogenesis, or organ formation. With all this, it has been developed not just a migration model but a workbench to investigate cell response to a wide variety of external stimuli. Furthermore, with its modular form, the model can be constantly updated and redefined as advancements are made in clarifying how cellular events take place.

## Supplementary Material

SupFile

## Figures and Tables

**Table 1:** Parameters for the fluid-chemical and mechanical analysis

Symbol	Variable	Value
Δ *P*	Pressure gradient at the microdevice	40 [Pa]
*D*	Diffusivity constant	10^−9^ [m^2^/s]^a^
* κ *	Gel permeability	10^−13^ [m^2^]^a^
* μ *	Fluid viscosity	10^3^ [Pa.s]^a^
* ρ *	Fluid density	10^3^ [kg/m^3^]^a^
Δ *C*	Chemical gradient	1 [mol/m^3^]
*K*_pas_	Passive resistance of cell cytoskeleton	1 [kPa]^b^
*K*_act_	Actin stiffness	10 [kPa]^b^
*ε*_max_, *ε*_min_	Maximum/minimum cell strain	−0.4,0.4^b^
$\sigma_{\max}^{\text{cort}}$	Maximum stress of the acto-myosin (AM) system at the cortex zone	2.5 [kPa]^b^
$\sigma_{\max}^{\text{cyto}}$	Maximum stress of the acto-myosin (AM) system at the cytoplasm	1.5 [kPa]

a [13],

b [22].

**Table 2:** Constant parameters of the probability functions

Symbol	Variable	Value
$p_{+}^{0},p_{-}^{0}$	Minimum probabilities of voxel addition/removal	0.1, 0.1
$p_{+}^{\max},p_{-}^{\max}$	Maximum probabilities of voxel addition/removal	0.8, 0.4
$k_{+}^{0},k_{-}^{0}$	Addition/removal rate	0.4, 0.4 [min^−1^]
$\lambda_{+}^{\sigma },\lambda_{-}^{\sigma }$	Sensitivity constants of addition/removal regarding cell stress magnitude	0.0035, 0.0035
$\lambda_{+}^{\Delta \sigma },\lambda_{-}^{\Delta \sigma }$	Sensitivity constants of addition/removal regarding cell stress gradient	0.004, 0.004
$\lambda_{+}^{C},\lambda_{-}^{C}$	Sensitivity constants of addition/removal regarding chemical concentration	0.3, 0.3
$\lambda_{+}^{F},\lambda_{-}^{F}$	Sensitivity constants of addition/removal regarding flow direction	0.004, 0.004
*dt*	Time step	5 [min]

## Abbreviations

FE: Finite elements
ECM: Extracellular matrix
CSK: Cytoskeleton
FEM: Finite element method
SFEM: Surface finite element method
MMPs: Matrix metalloproteinases
