# Supplementary material for: Probabilistic Voxel-Fe model for single cell motility in 3D
Source: In Silico Cell Tissue Sci. Author manuscript; Available in PMC 2015 Aug 17. (PMC4538854; doi:10.1186/2196-050X-1-2)
Supplement: SupFile [file NIHMS64308-supplement-SupFile.pdf]

Fluid-chemical simulation with cell body embedded in a porous ECM

To ensure that the body of a single cell embedded in a porous ECM does not affect the fluid and chemical fields of the whole microdevice, a specific analysis taking the cell geometry from a previous simulation of the migration model was performed. Since in this model the cell body is discretized with voxels the surface was smoothed and the resultant geometry was imported in COMSOL to perform the computation (Figure S1). Note that in this particular case each voxel is 1 μm^3 instead of 9 μm^3 as used in the main manuscript. Hence, the cell was ~20 μm length in the chosen frame.

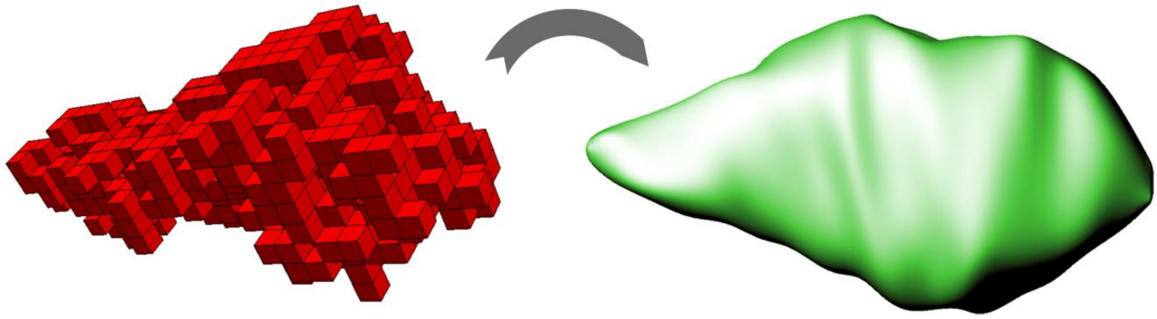

Figure S1: Cell geometry smoothing.
The voxelized-cell body from the migration model (left) is smoothed (right) to perform the fluid-chemical simulation.

For the analysis a cubical domain of 60×60×60 μm is used, and the smoothed cell geometry is embedded in the central part (Figure S2). Periodic conditions are established in lateral boundaries, and no-slip condition is considered at the cell surface. Speed-pressure inputs and porous material properties are taken from the microdevice simulation, choosing a zone of maximum fluid velocities to enhance the effects of the cell body. A tetrahedral mesh is used in the whole domain, with finer elements at the cell surface. As in the main microfluidic simulation, the steady-state solution is computed using the FEM software COMSOL.

The results show how both the speed magnitude and stream lines are slightly affected by the cell inclusion into the porous material (Figure S3A). Changes in flow velocity are only noticeable within 1-2 μm from the cell surface, a negligible distance compared with the dimensions of the whole collagen which are of the order of mm.

Similarly, chemical (Figure S3B) and pressure (Figure S4) gradients are practically unaffected, maintaining a linear increasing fashion with x-direction. In sum, this confirms the assumption that the cell body can be neglected in the simulation of the whole microdevice. Nevertheless, the slight changes occurring at the cell surface due to its geometry could be, in reality, critical for the cell to choose a migration direction. Such detailed considerations are, for the moment, out of scope of the model presented in the manuscript.

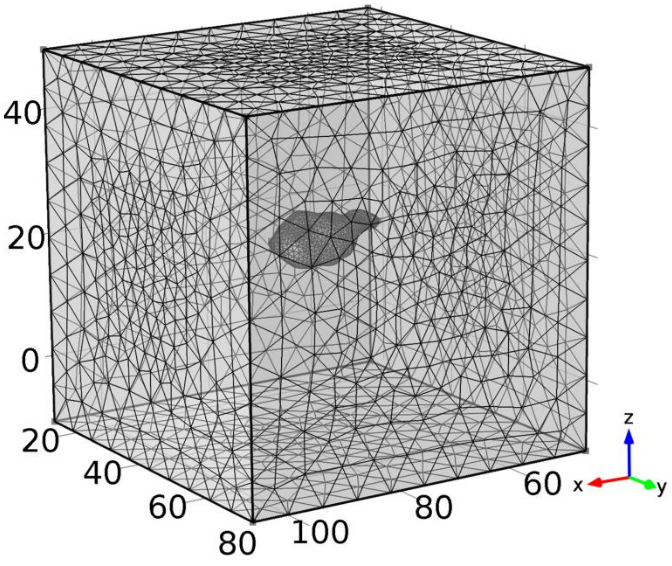

Figure S2: Tetrahedral mesh used in the simulations.
The cell body is embedded in a cubical domain of 60×60×60 µm. Finer elements are used at the cell surface.

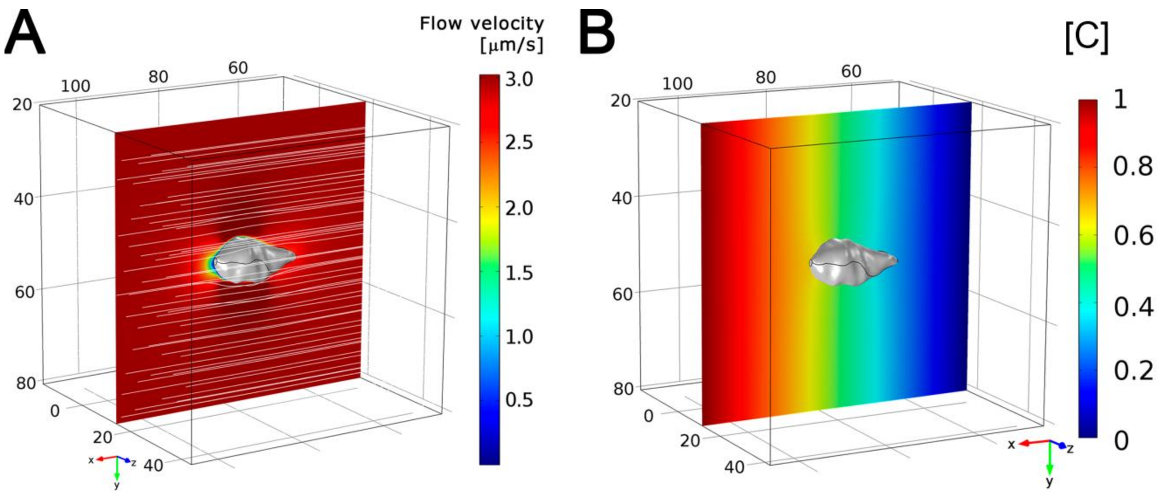

Figure S3: Flow velocity and ECM chemical concentration.
A) Flow velocity field and stream lines (white) around the embedded cell body (gray). Speeds are shown in a middle cut plane. B) Normalized chemical gradient along the ECM.

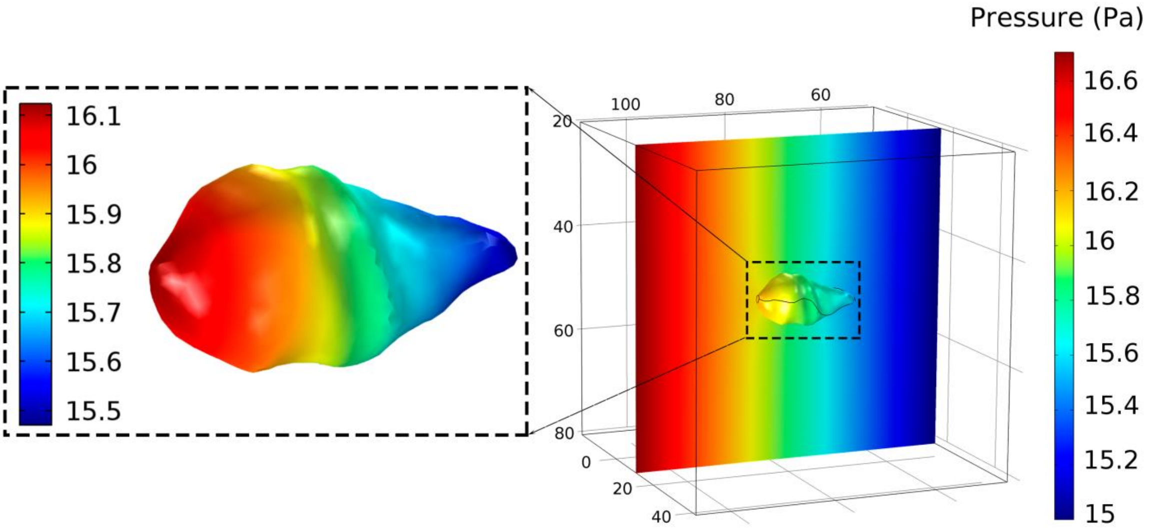

Figure S4: Pressure gradient along the ECM and cell surface.
The pressure changes linearly along the porous ECM, slightly affected by the cell body inclusion. Detail of pressure along cell surface is magnified.

Probability functions sensitivity analysis

A sensitivity analysis is performed to better understand the effect of each separate factor on the probability of adding/removing voxels:

$$p_+ = p_+^0 + p_+^{\max}\left(1 - e^{-k_+^0\left(\lambda_+^\sigma F_+^\sigma + \lambda_+^{\Delta\sigma} F_+^{\Delta\sigma} + \lambda_+^{\mathrm{C}} F^{\mathrm{C}} + \lambda_+^{\mathrm{F}} F_+^{\mathrm{F}}\right)dt}\right)$$

$$p_- = p_-^0 + p_-^{\max}\left(1 - e^{-k_-^0\left(\lambda_-^\sigma F_-^\sigma + \lambda_-^{\Delta\sigma} F_-^{\Delta\sigma} + \lambda_-^{\mathrm{C}} F^{\mathrm{C}} + \lambda_-^{\mathrm{F}} F_-^{\mathrm{F}}\right)dt}\right)$$

Due to the number of factors and high variety of combinations, σ_{v} is represented in the x-axis while varying other parameters. First, the number of factors involved and activated at the same time is explored. For this analysis all the factors have the same weights ($\lambda : \text{constant}$). In addition, to consider the maximum probability in each case, all the F 's depending on angles are considered aligned, that is, cosines and sines equal to 1. Figure S5 shows the probability of adding and removing voxels as a function of the cell stress and the active factors. In both cases, p_* increases exponentially with σ_{v} since F_*^σ has a linear dependence with cell stress. Adding new factors increases substantially the probabilities, but saturates when all the factors are acting at the same time (darker lines).

Secondly the effect of the alignment is tested. In particular, chemical and flow inputs are deactivated, taking only into account cell stress magnitude and gradient factors (F_+^{σ} and $F_+^{\Delta\sigma}$). In case of a voxel completely perpendicular with the maximum stress direction ($\cos(\theta)=0$ or $\sin(\theta)=0$ for adding or removing probabilities respectively), the exponent becomes null and hence $p_* = p_*^0$. As the alignment increases, so does the probability, more notably for higher stresses (Figure S6).

Next the effect of the sensitivity constants (λ) is analyzed. Specifically λ_*^{σ} is varied while $\lambda_*^{\Delta\sigma}$ is held constant and $\lambda_*^{\mathrm{C}} = \lambda_*^{\mathrm{F}} = 0$. Note that considering a perfect alignment ($\cos(\theta)=1$, $\sin(\theta)=1$ for adding/removing) and the control value of $\lambda_*^{\Delta\sigma}$ produces an offset of 0.3 and 0.15 (0.4 and 0.25 when adding p_*^0) in the probabilities of adding/removing voxels respectively, as can be seen in Figure S7. Due to this offset the functions are closer to saturation, explaining the minor changes produced in p_* (especially in p_-) for bigger variations of λ_*^{σ}. This is particularly important when all the factors are activated.

Finally the adding/removing rate (k_*^0) is varied. Since this parameter affects equally and at the same time to all the factors, small changes produce noticeable effects. These effects, however, are stronger for low rate values. For higher ones, the exponential function saturates producing very slight changes on the probability. Note that in Figure S8 the saturation is enhanced due to the consideration of perfect alignment (and therefore higher F values) as previously.

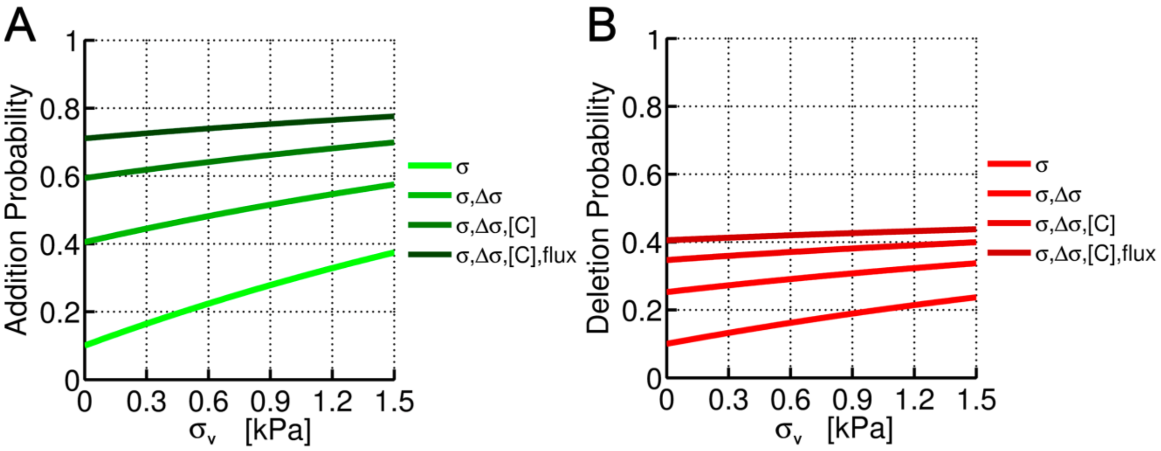

Figure S5: Effect of the number of factors on the probability functions.

A) Adding voxels probability. B) Removing voxels probability.

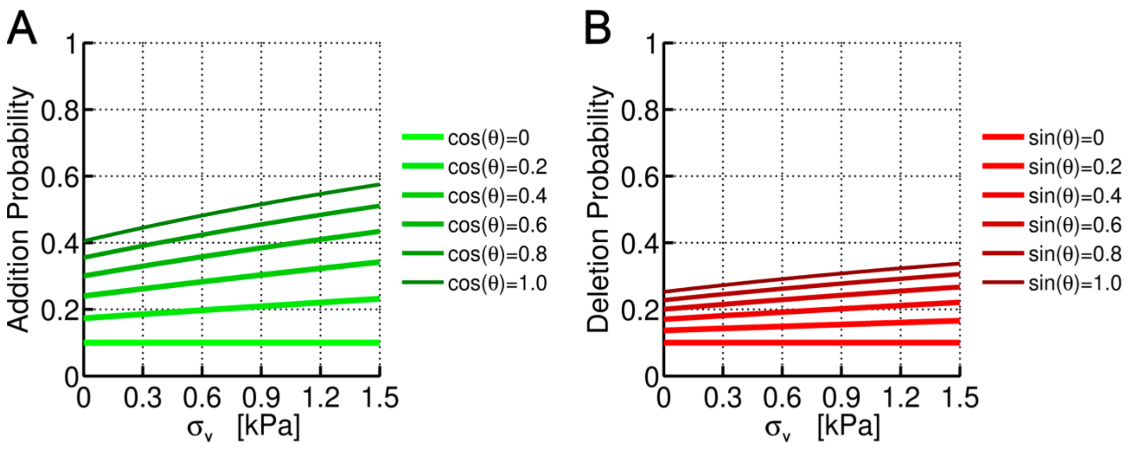

Figure S6: Effect of the alignment on the probability functions.

A) Adding voxels probability. B) Removing voxels probability.

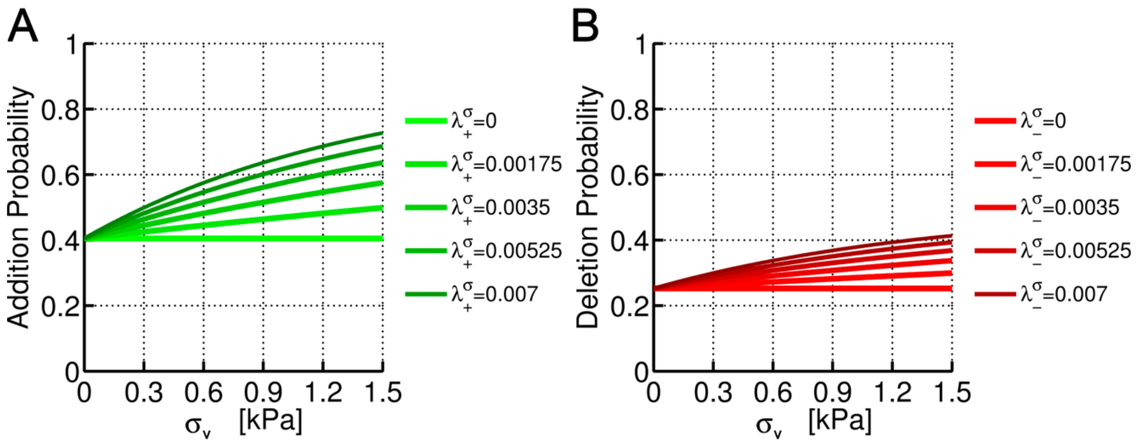

Figure S7: Effect of the sensitivity constants on the probability functions.

A) Adding voxels probability. B) Removing voxels probability.

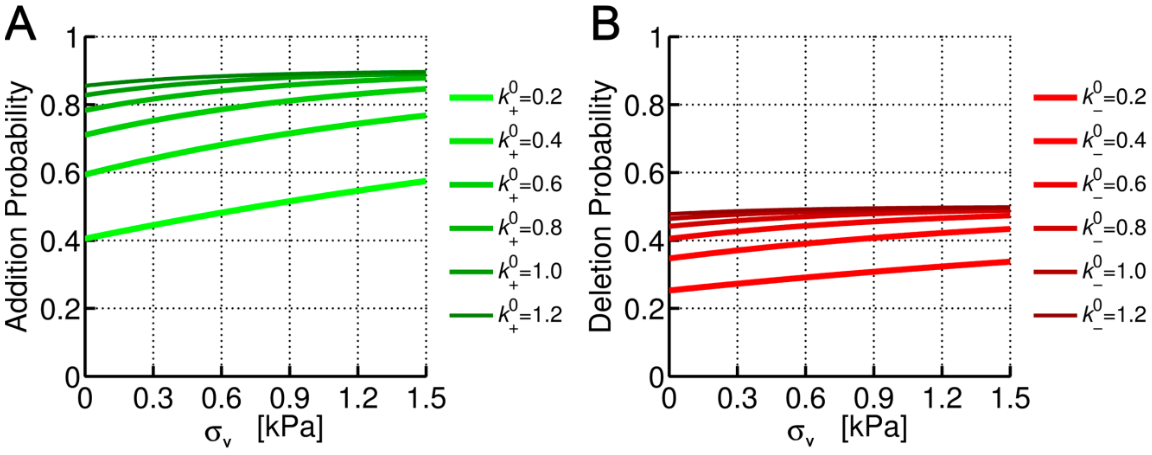

Figure S8: Effect of the adding/removing rate on the probability functions.

A) Adding voxels probability. B) Removing voxels probability.
